# Genome-wide association study of the reproductive, body size, and carcass-related latent and directly measured traits in admixed beef heifers

**DOI:** 10.3389/fgene.2026.1653878

**Published:** 2026-07-07

**Authors:** Muhammad Anas, Bin Zhao, Haipeng Yu, Carl R. Dahlen, Kendall C. Swanson, Kris A. Ringwall, Lauren L. Hulsman Hanna

**Affiliations:** 1 Department of Animal Sciences, North Dakota State University, Fargo, ND, United States; 2 Department of Statistics, North Dakota State University, Fargo, ND, United States; 3 Department of Animal Sciences, University of Florida, Gainesville, FL, United States; 4 Dickinson Research Extension Center, North Dakota State University, Dickinson, ND, United States

**Keywords:** Bayesian network learning, factor analysis, genome-wide association studies, latent variables, multi-trait model, structural equation model

## Abstract

Latent variables derived through factor analysis reveal the underlying biological traits (UBT) in organisms. However, research on UBT development and genomic applications in beef cattle is limited. This study aimed to model previously identified economically important UBT in genome-wide association studies (GWAS) using univariate and multivariate approaches. Data on 35 traits related to the body size, reproduction, and carcass characteristics from 297 admixed beef heifers were analyzed using two models. Due to the sample-size constraints, the two models utilized were: 1) all traits included (n = 161) and 2) optimized record numbers by segregating the reproductive and body size traits (n = 297) from carcass traits (n = 161). The UBT identified from a prior study included body size (BS) and body composition (BC) in model 1 and BS, ovary size (OS), and yield grade (YG) in model 2, along with non-contributing but economically relevant traits such as body density (DENS) and intra-muscular fat (IMF). Genotypically adjusted causal networks showed that BS influenced BC (model 1) and OS (model 2). Multi-trait and structural equation modeling (SEM) GWAS approaches were used to integrate BS with BC and OS, while univariate modeling was used for unrelated UBT and direct traits, such as YG, IMF, and DENS. Across all the approaches, 1,911 SNP from nine different regions were identified and mapped to 98 features, including genes, pseudogenes, and multiple non-coding and translational RNA. Enrichment analysis highlighted extracellular matrix-receptors, *HERC* family genes in cellular growth, desmosomes in morphogenesis, and energy metabolism related to *PIGY* genes. Genes linked to muscle and carcass traits, including *FAM184B, NCAPG*, and *LCORL*, were also identified. The heritability of UBT (0.46–0.84) was higher than that of individual traits, particularly for reproductive and carcass-related traits. This study provides insights into the relationships of body and carcass-oriented traits in admixed beef heifers that are directly relevant to phenotyping and genetic evaluations being conducted in the beef industry.

## 1 Introduction

The development of underlying biological traits (UBT), which are latent overarching variables representing a set of interrelated traits, is becoming more common due to advancements in phenotypic data collection technologies (Mccormick et al., 2016; Yu et al., 2019; Momen et al., 2021). The development of low-cost high-throughput genotyping technologies (Elshire et al., 2011; Kumar et al., 2012) has made livestock selection indispensable. However, precise animal selection also depends on accurate phenotyping to ensure efficient phenome-to-genome association (Daetwyler et al., 2008; Silva et al., 2021). Animal selection using UBT phenotypes is complex because no practical real-time biological measures are available to keep track of these traits except for the set of traits measured for the animals that were used to create these UBTs (Yu et al., 2020; Anas et al., 2025).

The contributing structure of the available traits to develop these complex UBTs can vary depending on the modeling approaches, such as the composite index, principal component, and factor analyses (Olasege et al., 2019). The composite index approach was used for body conformational and dairy capacity-associated traits in Holstein cows (Čítek et al., 2022), but since this approach does not capture the correlated nature of these traits, a multi-trait approach has also been adopted to compensate for its limitation (Lu et al., 2018). For morphological traits, however, principal component- and factor analyses-based approaches are preferred (Olasege et al., 2019). A limitation of principal component-based approaches is that the method tries to capture a set of parameters with the maximum variance, but those parameters (i.e., traits) can be opposite or antagonistic; therefore, those traits may not contribute to the developed latent variable at all (Yu et al., 2020; Yao and Ochoa, 2023).

Considering the given approaches, the factor analysis-based approach appears to be better for developing complex underlying biological phenotypes, and it has recently been adopted in dairy (Macciotta et al., 2012; Lurdes Kern et al., 2014) and dual-purpose cattle in China (Xu et al., 2022). It has also been proven effective in developing UBT phenotypes in beef cattle, such as temperament (Yu et al., 2020). Following this, the UBT phenotypes for the reproductive, body size, and carcass-related traits were developed for admixed beef heifers, and their causal network structure was identified using Bayesian network learning (BNL) in our recent study (Anas et al., 2025). The adjusted network structure in Anas et al. (2025) indicated that some UBTs indirectly influenced other traits, prompting us to explore the causal effect model or structural equation modeling (SEM) approaches, which have recently gained traction in dairy cattle research (Pegolo et al., 2020; 2021). Therefore, the objectives of this study are to effectively model these economically important UBTs and the independent traits found in Anas et al. (2025) in genome-wide association analysis (GWAA) to identify genomic regions of interest and compare the identified regions from univariate, multi-trait, and SEM approaches.

## 2 Materials and methods

### 2.1 Phenotypes and underlying biological phenotypes

Anas et al. (2025) described how the UBTs for this study were developed. Briefly, we started with yearling admixed beef heifers (n = 336) from the North Dakota State University (NDSU) Dickinson Research Extension Center. The admixed heifers consisted of the primary breed structure of 1) influenced (I, ≥50%) by the American Aberdeen (ADI; n = 59), Red Angus (ARI; n = 136), Angus (ANI, black Angus; n = 42), Gelbvieh (GV; n = 15), Simmental (SMI; n = 35), Shorthorn (SHI; n = 10), and Limousin (LMI; n = 4) and 2) the true first crosses of available purebreds (F_1_; n = 33). The heifer cohorts underwent a 105-day feeding trial at the NDSU Beef Cattle Research Complex as yearlings, and data were collected in the periconceptional period for heifers from 2015 to 2018. All reproductive ultrasound measures were collected before breeding from all heifers using the protocol by Cushman et al. (2009). The reproductive and body size-related parameters for all heifers were collected at the start of the trial. The body carcass ultrasound measures were collected using the available protocol (Wall et al., 2004) for heifers each year, but due to data loss, only data for heifers from 2015 to 2016 were available. Given considerations in Anas et al. (2025), there were 16 phenotypes used in this study. These phenotypes included 1) reproductive characteristics of ovary diameter (OD; left, LOD; right, ROD), uterine horn diameter (UHD), and antral follicle count (AFC); 2) body size measures of girth (heart, HG; mid, MG; flank, FG), body length (BL), hip (height, HH; width, HW), and density (DENS); and 3) carcass ultrasound measures of the ribeye area (REA), rib fat (RIB), intramuscular fat (IMF), rump fat (RMP), and yield grade (YG). Given the loss of data in carcass ultrasound measures, two models (models 1 and 2) were utilized, following the recommendations of Anas et al. (2025). Model 1 consisted of heifers with all the phenotype data available (n = 161), whereas model 2 maximized the sample size by breaking heifer data into reproductive and body size (n = 297) and carcass ultrasound (n = 161) datasets. These datasets and sample sizes included prior filtering in Anas et al. (2025) for missing samples, outliers, and correlation among all the available phenotypes so that the phenotypes used in this study were the best representative of the type (reproductive, body size, and carcass) and heifers available.

**FIGURE 1:**
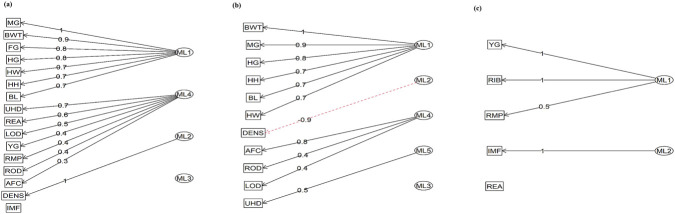
Factor analysis results on the available phenotypes from Anas et al. (2025) that was used to develop the underlying biological traits (UBTs) for **(a)** model 1 with all phenotypes as one dataset (n = 161 heifers), leading to the UBT of body size (ML1) and body composition (ML4); **(b)** model 2 with phenotypes of body size and reproductive ultrasound measures (n = 297 heifers) leading to the UBT of body size (ML1) and ovary size (ML4); and **(c)** model 2 with phenotypes of carcass ultrasound measures (n = 161 heifers) leading to the UBT of yield grade (ML1). The phenotypes included antral follicle count (AFC), body density (DENS), body length (BL), body weight (BWT), flank girth (FG), heart girth (HG), hip height (HH), hip width (HW), intra-muscular fat (IMF), left ovary diameter (LOD), mid girth (MG), ribeye area (REA), right ovary diameter (ROD), rump fat (RMP), uterine horn diameter (UHD), and yield grade (YG).

In Anas et al. (2025), exploratory factor analysis (EFA) was used to identify the hidden latent variables and structure for both models using the R v4.1.3 package *psych* v2.3.3 (Revelle, 2017; see Figure 1). The sample adequacy of 0.5 or higher for the Kaiser–Meyer–Olkin (KMO) criterion was considered sufficient (Kaiser, 1958) to move forward with confirmatory factor analysis (CFA). For CFA, the *blavaan* v0.4-7 (Merkle and Rosseel, 2015) R package was used with default priors to set the posterior mean values as the UBT phenotype for the identified latent variables. Based on Anas et al. (2025), model 1 (KMO of 0.63) UBT phenotypes included body size (BS) and body composition (BC), with a 0.5 directional signal of BS contributing to BC, while model 2 (KMO of 0.57 and 0.59, respectively) UBT phenotypes included BS, ovary size (OS), and yield grade (YG UBT), with a 0.5 directional signal of BS contributing to OS. Although these are weaker signals and the sample size is small, the aim remained to study the different statistical approaches on these UBTs and identify the biological regions of interest using GWAA approaches. Figure 1 illustrates how direct phenotypes relate to UBT phenotypes for this study. These outcomes established the UBT phenotypes that were used in this study for GWAA using univariate, multi-trait, and structural equation modeling (see Section 2.2). Body density (DENS) and intra-muscular fat (IMF) did not group with any of the UBT phenotypes, thereby classifying them as non-contributing but economically relevant traits (NC–ERT) that were also included in univariate GWAA of this study and in comparison to other traits. Being mindful of the ease of producer implementation and the application of this approach in the field for the selection of beef cattle, we also selected directly measured traits with the highest loading for each UBT phenotype in model 2 (which maximizes the sample size) as representative direct traits in univariate GWAA. The representative traits (Figure 1) included BWT (highest loading for BS), AFC (OS), and YG (YG UBT). The results from these representative traits’ GWAA were compared to the univariate and multi-trait UBT outcomes.

### 2.2 Genomic data filtering and association analysis

The initial population of animals (n = 336) was genotyped using the GeneSeek Genomic Profiler 150K for Beef Cattle (Neogen GeneSeek, Inc., Lincoln, NE), and the quality was checked (QC; Bhowmik et al., 2023; Anas et al., 2025). Briefly, genotyping led to 138,893 SNP markers, but only 132,368 autosomal SNP markers were further analyzed for this study. The QC criteria of minor allele frequency (MAF) threshold of 5%, call rate of markers more than 95%, and an exact Hardy–Weinberg equilibrium (*P* < 0.0001) (Wigginton et al., 2005) led to 117,373 SNP markers. All QC analyses were conducted through R v4.1.3, and markers were retained for further sub-setting based on the model and available samples (i.e., model 1 or model 2 approaches). Since models 1 and 2 differed in the animals involved, each model subset was rechecked to ensure consistency in the use for MAF of 5% and call rate of 95% with snpReady v0.9.7 (Granato et al., 2018), leading to 115,388 markers for downstream GWAA of UBTs of both the models (1 and 2), NC–ERT, and the representative traits. Bhowmik et al. (2023) used this population with other cattle that included this marker panel to explore the genetic diversity and linkage disequilibrium (LD) levels using this same marker panel, finding that ample LD (r^2^ > 0.1) exists among this dense marker panel, thus providing adequate statistical power to detect the genomic regions of larger effect in this population (e.g., see Gondro et al., 2013).

All GWA analyses (n = 14 models) were conducted using the JWAS package v2.3.0 of Julia v1.12.3 (Cheng et al., 2018) and a window size of 1 MB to reduce issues related to linkage disequilibrium. Fixed systematic effects that were modeled included the intercept, birth year (year; n = 2 or 4 given the phenotypes used), dam’s age (n = 7), and PBG (n = 8 or 9 given the phenotypes used). The animals were treated as a random effect with known pedigree. The current study’s sample size was too small to estimate π, the proportion of markers with zero effect, given the amount of SNP markers available (e.g., Wolfe et al., 2017). Therefore, the BayesB method was selected to allow for π and individual effects per marker to be estimated (Habier et al., 2011). In all cases, 2,000 Markov Chain Monte Carlo (MCMC) samples were saved per analysis. To avoid autocorrelation and larger effective sample sizes (Gelman et al., 2013), thinning was carried out every 100 samples, and the first 10,000 iterations were discarded as burn-in. This led to 210,000 MCMC iterations per analysis. Saved samples were then used in the *GWAS* function of the JWAS package to estimate the posterior genetic variance and window posterior probability of association (PPA_W_). The significance of the regions was identified based on PPA_W_ of 0.8 or higher (Fernando et al., 2017).

All phenotypes (UBTs, NC–ERT, and representative traits) underwent univariate analyses with π = 0.001 (almost all markers used), 0.90 (10% of markers included), and 0.95 (5% of markers included). The UBTs also underwent 1) multi-trait modeling (MTM) that did not include a pre-defined correlation structure and 2) a structure equation modeling (SEM) that did include a pre-defined correlation structure given the BNL outcomes from Anas et al. (2025). Due to the increased computational time of MTM and SEM analyses and the current sample size of animals, only π = 0.90 was analyzed for comparison to univariate analyses. Across analyses, MCMC samples were combined and summarized using R software. Diagnostics and summaries of MCMC samples were analyzed using the R software *coda* package (Plummer et al., 2006), including the effective sample sizes, autocorrelation, and visual assessment of sample trace and density plots. The script used for all GWAA, including the correlation structure in Julia and post-processing in R, is provided in Supplementary Figure S1.

The associated windows were mapped manually to nearby associated features (i.e., genes, pseudogenes, long non-coding RNAs, micro-RNAs, small non-coding RNAs, and tRNAs) based on ARS-UCD 1.2 assembly (Rosen et al., 2020), following the marker panel map. Functional enrichment analysis was conducted using the *Bos taurus* assembly (ARS-UCD 1.2, Rosen et al., 2020) and g:Profiler (v. e114_eg62_p19_27110d83) with g:SCS multiple testing correction and the application of a significance threshold of 0.05 (Kolberg et al., 2023).

## 3 Results

### 3.1 MCMC diagnostics summary

All MCMC samples of the variance parameters estimated, effective sample sizes, and autocorrelation calculated from these MCMC samples are available in Supplementary Figure S2. Based on these MCMC samples saved (n = 2,000), the effective sample sizes ranged from 323.54 to 1,777.24 across all the models and chosen π. When considering π = 0.90, effective sample sizes ranged from 359.59 to 1,667.77 across all the models. The effective sample sizes were lower for OS and AFC, which was likely due to the stair-step nature of AFC records (interval nature and not fully continuous), and the carcass traits due to the lower sample sizes. Nevertheless, model 1 UBTs had larger effective sample sizes than the carcass-only traits, even in MTM and SEM analyses. When considering π = 0.90, autocorrelation was less than 0.20, 0.11, 0.07, and 0.04 by 5, 10, 50, and 100 samples, respectively, across all the models. All analyses yielded over 300 effective samples and reasonably low autocorrelation based on the thinning used, indicating that the posterior summaries were reasonable estimates of true values. Visual assessment of trace and density plots also confirmed proper mixing and posterior distributions of parameters (plots not shown due to the number but can be replicated using supplementary data and scripts).

### 3.2 Heritability, genetic correlation, and causal structure estimates

**FIGURE 2:**
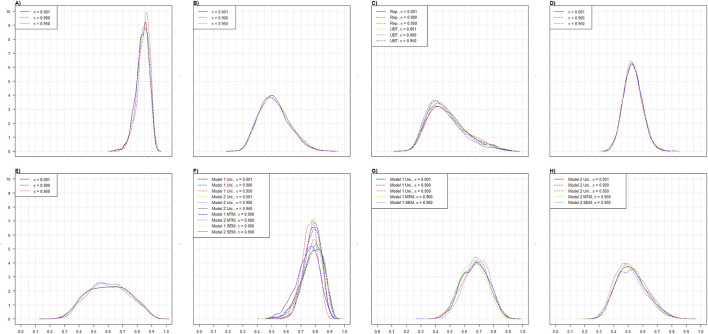
Posterior densities of heritability Markov Chain Monte Carlo samples saved (n = 2,000) for **(A)** body weight; **(B)** antral follicle count; **(C)** representative (rep.) and underlying biological trait (UBT) yield grade; **(D)** body density; **(E)** intra-muscular fat; **(F)** body size analyzed in univariate (uni.), multi-trait (MTM), and structural equation modeling (SEM) approaches across models 1 and 2; **(G)** body composition analyzed in uni., MTM, and SEM approaches, and **(H)** ovary size analyzed in uni., MTM, and SEM approaches when considering the different levels of π, the proportion of markers with zero effect. Model 2 maximized the sample size by breaking the heifer data into reproductive and body size (n = 297) and carcass ultrasound (n = 161) datasets.

**FIGURE 3:**
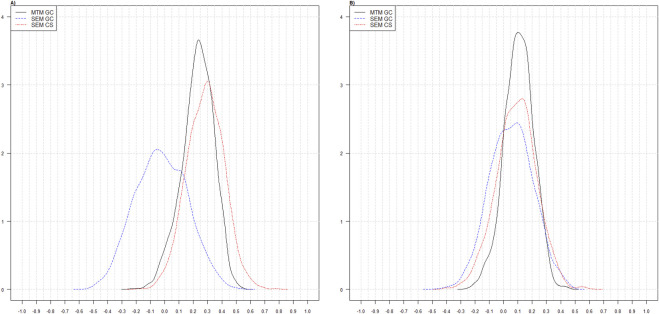
Posterior densities of genetic correlation (GC) and causal structure (CS) Markov Chain Monte Carlo samples saved (n = 2,000) for multi-trait (MTM) and structural equation modeling (SEM) analyses of **(A)** model 1 underlying biological traits (UBTs) and **(B)** model 2 UBTs when π, the proportion of markers with zero effect, was set to 0.90. Model 2 maximized the sample size by breaking the heifer data into reproductive and body size (n = 297) and carcass ultrasound (n = 161) datasets.

Posterior density plots of heritability ( ${\hat{h}}^{2}$ ) per trait across analyses and chosen π are provided in Figure 2, and genetic correlations ( ${\hat{r}}_{g}^{2}$ ) and causal structures when π = 0.90 are provided in Figure 3. Focusing on π = 0.90, the posterior estimates, credible intervals, and highest posterior densities per trait and analysis are provided in Table 1, with other π estimates provided in Supplementary Figure S2. Regardless of the approach, when π = 0.90, ${\hat{h}}^{2}$ ranged from 0.4593 ± 0.1206 to 0.8398 ± 0.0439, where UBT YG and YG were the least heritable traits and BWT was the most heritable, followed by BS and BC (Table 1). Genetic correlations were weak for SEM analyses and moderately weak for MTM analyses, where all four analyses had credible intervals and the highest posterior density overlapping zero (Table 1). Causal structures in SEM analyses were also weak, with only model 1 having a causal structure that did not overlap zero, although the lower bound was very close to zero (Table 1). The initial directional signal of 0.5 for SEM was weak, and with the lower sample size, it is not unexpected that these parameters were close to zero if not zero.

**TABLE 1:** Posterior heritability estimates ( ${\hat{h}}^{2}$ ), genetic correlations ( ${\hat{r}}_{g}^{2}$ ), and causal structure (CS) estimates with standard deviation (SD) and 95% credible interval (CI) and the highest posterior density (HPD) using BayesB with π = 0.90 and univariate, multi-trait, and structural equation modeling approaches for the underlying biological traits (UBTs), representative (rep.) traits, and non-contributing but economically relevant traits (NC–ERT).

Analysis^1^	Trait category^2^	Trait^3^	Mean ± SD	CI	HPD
Univariate	Model 1 UBT	BS	0.7794 ± 0.0756	0.6054–0.8963	0.6219–0.9032
​	​	BC	0.6661 ± 0.0915	0.4913–0.8371	0.5016–0.8419
​	Model 2 UBT	BS	0.7782 ± 0.0574	0.6541–0.8772	0.6623–0.8817
​	​	OS	0.5300 ± 0.1046	0.3510–0.7242	0.3395–0.7242
​	​	YG	0.4694 ± 0.1225	0.2879–0.7565	0.2701–0.7359
​	Rep. traits	BWT	0.8398 ± 0.0439	0.7429–0.9109	0.7525–0.9171
​	​	AFC	0.5191 ± 0.1028	0.3467–0.7347	0.3371–0.7226
​	​	YG	0.4593 ± 0.1206	0.2864–0.7640	0.2701–0.7359
​	NC–ERT	DENS	0.5328 ± 0.0628	0.4175–0.6583	0.4213–0.6616
​	​	IMF	0.6078 ± 0.1340	0.3659–0.8590	0.3700–0.8610
MTM	Model 1	BS	0.7444 ± 0.0787	0.5732–0.8756	0.5819–0.8816
​	​	BC	0.6554 ± 0.0892	0.4779–0.8208	0.4882–0.8292
​	​	${\hat{r}}_{g}^{2}$	0.2297 ± 0.1137	−0.019–0.4226	−0.0045–0.4308
​	Model 2	BS	0.7595 ± 0.0561	0.6365–0.8546	0.6481–0.8629
​	​	OS	0.5124 ± 0.1013	0.3391–0.7286	0.3395–0.7242
​	​	${\hat{r}}_{g}^{2}$	0.1062 ± 0.1038	−0.1174–0.2990	−0.0912–0.3166
SEM	Model 1	BS	0.7790 ± 0.0757	0.6061–0.8984	0.6297–0.9082
​	​	BC	0.6846 ± 0.0852	0.5089–0.8369	0.5223–0.8439
​	​	${\hat{r}}_{g}^{2}$	−0.0250 ± 0.1809	−0.3628–0.3263	−0.3752–0.3110
​	​	CS	0.2837 ± 0.1304	0.0282–0.5349	0.0260–0.5291
​	Model 2	BS	0.7795 ± 0.0582	0.6586–0.8772	0.6661–0.8820
​	​	OS	0.5098 ± 0.1032	0.3395–0.7348	0.3319–0.7202
​	​	${\hat{r}}_{g}^{2}$	0.0539 ± 0.1522	−02429–0.3588	−0.2667–0.3257
​	​	CS	0.0888 ± 0.1422	−0.1957–0.3588	−0.1957–0.3595

1 Multi-trait (MTM) and structural equation modeling (SEM) estimated pi as a dictionary based on individual traits, traits together (both), or neither trait. All estimates are reported based on their alignment.

2 Model 1 consisted of heifers with all phenotype data available (n = 161), whereas model 2 maximized the sample size by breaking heifer data into reproductive and body size (n = 297) and carcass ultrasound (n = 161) datasets for underlying biological traits (UBTs).

3 Trait abbreviations used include BS, body size; BC, body composition; OS, ovary size; YG, yield grade; BWT, body weight; AFC, antral follicle count; IMF, intra-muscular fat.

### 3.3 Genome-wide association and pathway analyses

**FIGURE 4:**
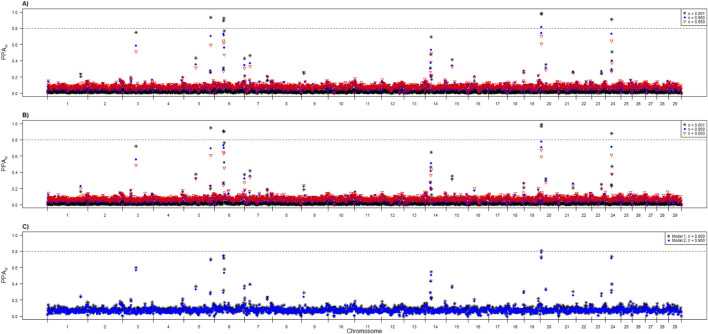
Posterior probability of association per window (PPA_w_) across the genome (n = 2,501 windows) for the underlying biological traits of **(A)** model 1 body size (BS) univariate, **(B)** model 2 BS univariate, and **(C)** multi-trait model (MTM) of BS for models 1 and 2 considering different levels of π, which is the proportion of genomic markers with zero effect. Model 2 maximized the sample size by breaking heifer data into reproductive and body size (n = 297) and carcass ultrasound (n = 161) datasets. A threshold of PPA_w_ ≥ 0.8 is shown with a dashed line.

**FIGURE 5:**
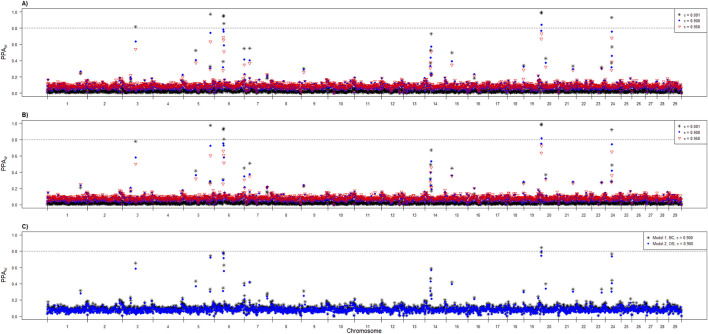
Posterior probability of association per window (PPA_w_) across the genome (n = 2,501 windows) for the underlying biological traits of **(A)** model 1 body composition univariate, **(B)** model 2 ovary size, and **(C)** multi-trait model (MTM) of model 1 BC and model 2 OS considering different levels of π, which is the proportion of genomic markers with zero effect. Model 2 maximized the sample size by breaking heifer data into reproductive and body size (n = 297) and carcass ultrasound (n = 161) datasets. A threshold of PPA_w_ ≥ 0.8 is shown with a dashed line.

**FIGURE 6:**
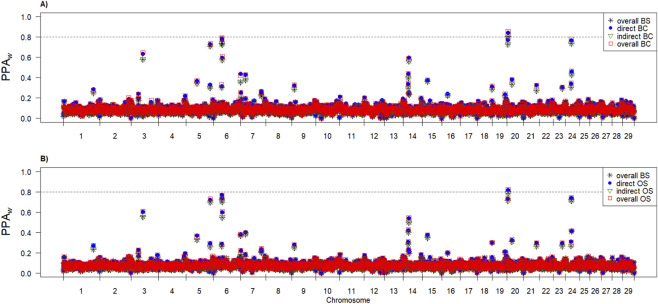
Posterior probability of association per window (PPA_w_) across the genome (n = 2,501 windows) for the underlying biological traits of structural equation model analysis for **(A)** model 1, where body size (BS) contributes to body composition (BC), and **(B)** model 2, where BS contributes to ovary size (OS). The direct, indirect, and overall probabilities are reported for BC and OS. Model 2 maximized the sample size by breaking heifer data into reproductive and body size (n = 297) and carcass ultrasound (n = 161) datasets. A threshold of PPA_w_ ≥ 0.8 is shown with a dashed line.

**FIGURE 7:**
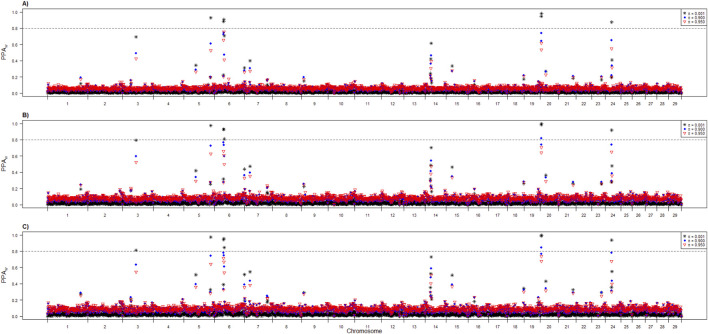
Posterior probability of association per window (PPA_w_) across the genome (n = 2,501 windows) for univariate analyses of the representative traits **(A)** body weight, **(B)** antral follicle count, and **(C)** yield grade considering different levels of π, which is the proportion of genomic markers with zero effect. The traits used model 2 population, which maximized the sample size by breaking heifer data into reproductive and body size (n = 297) and carcass ultrasound (n = 161) datasets. A threshold of PPA_w_ ≥ 0.8 is shown with a dashed line.

**FIGURE 8:**
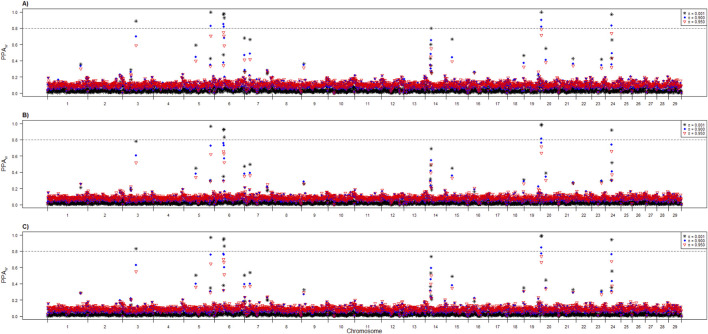
Posterior probability of association per window (PPA_w_) across the genome (n = 2,501 windows) for univariate analyses of the non-contributing yet economically relevant traits **(A)** body density and **(B)** intramuscular fat and **(C)** model 2 underlying biological trait yield grade considering different levels of π, which is the proportion of genomic markers with zero effect. The traits used model 2 population, which maximized the sample size by breaking heifer data into reproductive and body size (n = 297) and carcass ultrasound (n = 161) datasets. A threshold of PPA_w_ ≥ 0.8 is shown with a dashed line.

**FIGURE 9:**
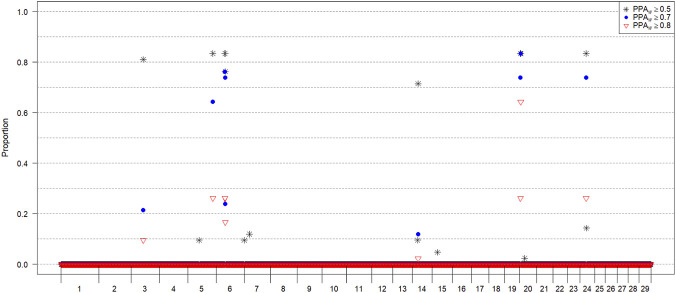
Proportion of models for which a specific window met the different levels of window posterior probability of association (PPA_w_) thresholds. The total models (n = 42) included 1) 10 univariate analyses when π, the proportion of markers with zero effect, was set to 0.001; 2) 10 univariate, four multi-trait, and eight structural equation analyses when π = 0.90; and 3) 10 univariate analyses when π = 0.95. Analyses represent both models 1 and 2, where model 2 maximized the sample size by breaking heifer data into reproductive and body size (n = 297) and carcass ultrasound (n = 161) datasets.

Posterior estimates and summaries of genomic windows across all analyses are available in Supplementary Figure S3. The PPA_W_ per window by BS UBTs univariate and MTM (Figure 4), BC or OS UBTs univariate and MTM (Figure 5), model 1 and model 2 SEM (Figure 6), and univariate representative (Figure 7), and NC–ERT and UBT YG traits (Figure 8) identified consistent regions of interest (Figure 9). A total of nine genomic regions (Table 2; Figure 9) were significantly associated (PPA_W_ ≥ 0.7 or 0.8) with at least one of the trait models across the chosen π. Of those nine genomic regions, six were consistently identified across different π for at least one trait, regardless of the GWAA approach. When allowing PPA_W_ ≥ 0.70 due to the smaller sample size, the percentage of genetic variance captured by the nine windows ranged from 0.1283% to 4.9175%. Averaging the percentage of genetic variance across a window for regions with PPA_W_ ≥ 0.70 led to a range of 0.1555%–0.4158%. Within these nine genomic windows, there were 1,911 SNPs.

**TABLE 2:** Genome-wide associated regions meeting the window posterior probability of association (PPA_W_ ≥ 0.7 or 0.8) threshold for univariate, multi-trait (MTM), and structural equation modeling (SEM) approaches of all the traits and chosen π.^1^

​	​	​	​	Traits		
BTA	Mb	No. SNP	Perc. Genet. Var	π = 0.001	π = 0.90	π = 0.95
3	52	193	0.1555	AFC, BS1, BS2, BC, DENS, IMF, YG, OS, and UBT YG	--	--
5	106	212	0.1770	AFC, BS1, BS2, BC, BWT, DENS, IMF, YG, OS, and UBT YG	AFC, BS1, BC, **DENS**, IMF, YG, MTM1 BS, MTM1 BC, MTM2 OS, OS, SEM1 BS, SEM1 BC, SEM2 OS, and UBT YG	DENS
6	36	245	0.2300	AFC, BS1, BS2, BC, BWT, DENS, IMF, YG, OS, and UBT YG	AFC, BS1, BS2, BC, BWT, **DENS**, IMF, YG, MTM1 BS, MTM1 BC, MTM2 BS, MTM2 OS, OS, SEM1 BS, SEM1 BC, SEM2 BS, SEM2 OS, and UBT YG	DENS, YG, and UBT YG
6	37	225	0.4158	AFC, BS1, BS2, BC, BWT, DENS, IMF, YG, OS, and UBT YG	AFC, BS1, BC, BWT, **DENS**, IMF, YG, MTM1 BS, MTM1 BC, MTM2 BS, MTM2 OS, OS, SEM1 BS, SEM1 BC, SEM2 BS, SEM2 OS, and UBT YG	BWT and DENS
6	38	170	0.1479	AFC, BS1, BS2, BC, BWT, DENS, IMF, YG, OS, and UBT YG	--	--
14	24	173	0.1569	AFC, BC, DENS, YG, and UBT YG	--	--
20	4	221	0.1826	AFC, BS1, BS2, BC, BWT, DENS, IMF, YG, OS, and UBT YG	AFC, BS1, BS2, BC, **DENS**, IMF, YG, MTM1 BS, MTM1 BC, MTM2 BS, MTM2 OS, OS, SEM1 BS, SEM1 BC, SEM2 BS, SEM2 OS, and UBT YG	DENS
20	5	240	0.2078	AFC, BS1, BS2, BC, BWT, DENS, IMF, YG, OS, and UBT YG	**AFC**, **BS1**, BS2, **BC**, BWT, **DENS**, **IMF**, **YG**, **MTM1 BS**, **MTM1 BC**, MTM2 BS, **MTM2 OS**, **OS**, **SEM1 BS**, **SEM1 BC**, SEM2 BS, **SEM2 OS**, and **UBT YG**	AFC, BS1, BC, DENS, IMF, YG, OS, and UBT YG
24	26	232	0.1932	AFC, BS1, BS2, BC, BWT, DENS, IMF, YG, OS, and UBT YG	AFC, BS1, BS2, BC, **DENS**, IMF, YG, MTM1 BS, MTM1 BC, MTM2 BS, MTM2 OS, OS, SEM1 BS, SEM1 BC, SEM2 BS, SEM2 OS, and UBT YG	DENS
Total	​	1,911	​	9	6	6

1 Model 1 (1) consisted of heifers with all phenotype data available (n = 161), whereas model 2 (2) maximized the sample size by breaking heifer data into reproductive and body size (n = 297) and carcass ultrasound (n = 161) datasets. Abbreviations include BTA, *Bos taurus* autosome, Mb, megabase window; MTM, multi-trait model; SEM, structure equation model; UBT, underlying biological trait; BS, body size; BC, body composition; OS, ovary size; YG, yield grade; IMF, intra-muscular fat; BWT, body weight; AFC, antral follicle count, No. SNP, number of single nucleotide polymorphism markers in that chromosomal window; Perc. Genet. Var., average percentage of genetic variance for that window if the PPA_*W*_ analysis threshold was 0.7 or higher. For SEM, the associated regions are reported for direct (d), indirect (id), and overall (o) based on the correlation structure of the UBTs involved. Traits are **bold** if PPA_*W*_ was 0.8 or higher.

**FIGURE 10:**
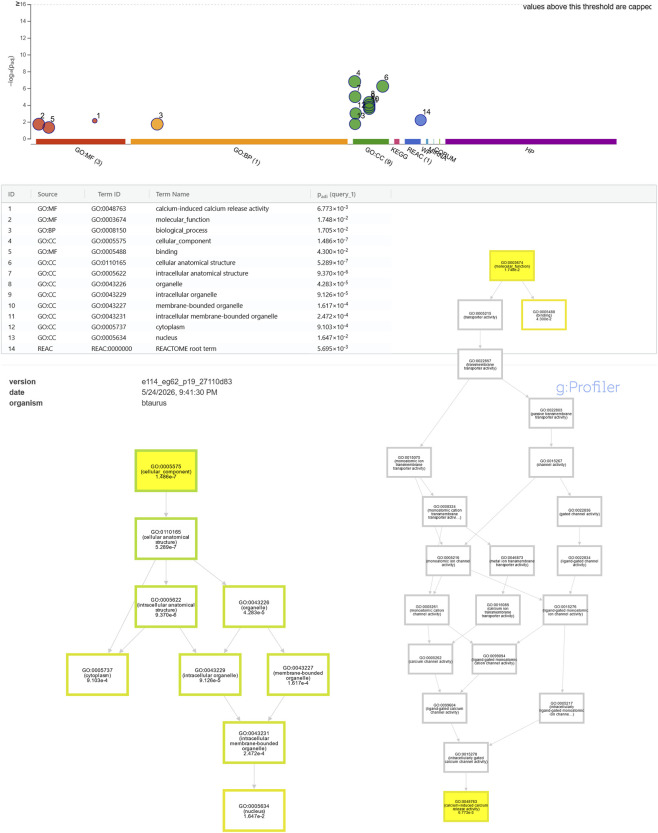
Functional enrichment analysis of significant outcomes from g:Profiler. Sources include Gene Ontology (GO), KEGG pathways, reactome (REAC), and WikiPathways (WP). The bubble size indicates the number of features involved, where larger bubbles represent more features. Only significant (g:SCS threshold of 0.05 or less) terms are numbered. Significant (yellow) molecular function cellular component terms with g:SCS values are shown as they progress with other significant and non-significant GO terms.

The features (n = 98) identified in these significant regions included genes, pseudogenes, non-coding RNA (long, small nuclear, and micro), and translational RNA (ribosomal and transfer) (Table 3). Functional enrichment analysis of these 98 features identified 13 significant Gene Ontology (GO) enrichments (Figure 10; Supplementary Figure S4). The number of features involved in these enrichments ranged from two to 51, with 12 enrichments having 25 or more features involved. The biological process GO enrichment only found the root term significant, indicating that many of the analyzed features are involved in biological processes, but it did not identify a specific process significantly. Most GO enrichments were found in cellular components leading to the involvement of the cytoplasm and nucleus, along with molecular functions leading to calcium ion binding (Figure 10). In addition, there were 24 features that aligned with the reactome database, but, similar to the biological process GO, the features did not identify a specific reactome term significantly.

**TABLE 3:** Genome-wide associated features located within regions with window posterior probability of association (PPA_W_ ≥ 0.8) for univariate, multi-trait, and structural equation model approaches.^1^

BTA	Mb	Genes	Pseudogenes	Translational RNA		Non-coding (nc) RNA			No. of features
				tRNA	rRNA	Long ncRNA	miRNA	snRNA	
3	52	*BARHL2 and ZNF644*	*LOC614424, LOC100140881, and LOC112445992*	*TRNAC-GCA*	​	*LOC112445877, LOC112445878, and LOC112445879*	*MIR2285B-2*	​	10
5	106	*CRACR2A, DDX11, FOXM1, FKBP4, ITFG2, NRIP2, PARP11, PRMT8, RHNO1, TEAD4, TEX52, TSPAN9, TSPAN11, and TULP3*	*LOC101902968*	​	​	*LOC100847686, LOC104972568, LOC104972569, LOC107132524, and LOC112446764*	​	​	20
6	36	*ABCG2, HERC3, HERC5, HERC6, IBSP, MEPE, NAP1L5, PIGY, PKD2, PPM1K, PYURF, and SPP1*	*LOC781421*	​	​	*LOC100847719, LOC104972724, and LOC112447053*	​	*LOC112447205*	17
6	37	*DCAF16, FAM184B, LAP3, LCORL, MED28, and NCAPG*	​	*TRNAA-CGC*	​	*LOC112447150*	​	​	8
6	38	​	*LOC782905*	​	​	​	​	​	1
14	24	*CYP7A1, FAM110B, LOC101902490, NSMAF, SDCBP, and UBXN2B*	​	*TRNAG-CCC*	​	*LOC107133116 and LOC112449508*	​	*LOC112449629*	10
20	4	*ATP6V0E1, BNIP1, CREBRF, DUSP1, ERGIC1, NEURL1B, NKX2-5, RPL26L1, and SH3PXD2B*	*LOC112443076*	*TRNAG-CCC*	*LOC112443038*	*LOC104975190, LOC104975192, LOC112442979, and LOC112442980*	​	​	16
20	5	*BOD1, C20H5orf47, CPEB4, NSG2, and STC2*	*LOC613880, LOC782443, LOC783392, and LOC104975196*	​	​	*LOC101909754, LOC104976639, LOC104976640, LOC107131404, and LOC112442981*	​	​	14
24	26	*DSC2 and DSC3*	​	​	​	​	​	​	2
No. Features	​	56	11	4	1	23	1	2	98

1 Abbreviations include BTA, *Bos taurus* autosome, Mb, megabase; RNA, ribonucleic acid; feature names follow the standard gene symbol for *Bos taurus*.

## 4 Discussion

Efficient selection for the key underlying biological traits of economic importance requires the identification of truly associated regions with effective sample size and high heritability (Wang et al., 2005). The UBTs developed in this study were either biologically immeasurable, i.e., body size (An et al., 2020), or had low-to-moderate heritability, i.e., reproductive traits (Cushman et al., 2014) and carcass traits (Rafter et al., 2021), in cattle. Factor analysis and adjusted BNL structure provided an opportunity to reveal the structure of the phenotypic parameters contributing to UBTs and their inter-relationship. The authors recognize that the sample size in this study was small. Given the generation interval of cattle and the expense of capturing such comprehensive datasets on a large scale, the findings of this study are relevant in helping guide future work and biological understanding. Our results (Table 1) showed that the heritability of reproductive, carcass-, and even body size-related traits improved compared to the heritability estimates of other individually measured traits from previous studies (Cushman et al., 2014; Rafter et al., 2021; Rostamzadeh Mahdabi et al., 2023). Despite the lower sample size of model 1, SEM was still able to estimate a non-zero causal structure, indicating a more relevant interaction of those UBTs than in model 2 (Table 1). Considering the representative traits, the heritability of BWT, AFC, and the initial yield grade were similar to that of their respective UBTs and higher than that shown in previously mentioned studies (Cushman et al., 2014; Rafter et al., 2021; Rostamzadeh Mahdabi et al., 2023). Furthermore, DENS, calculated as the body weight divided by body volume (L) (Anas et al., 2025), was consistently a driver of identified regions regardless of the chosen π (Table 2). Considering the traits involved in each UBT (Figure 1) and the representative and NC–ERTs used, the pairing of DENS with the given traits for a region likely signifies aspects of the trait that the genes are involved in. Very little literature is present regarding the genomic regions for DENS or the related attributes (e.g., Abo-Ismail et al., 2017; Ruchay et al., 2022).

The higher heritability estimates observed in this study may be a result of the GWAA approach (BayesB), the diverse nature of the population for these attributes, and due to the enhanced modeling of systematic effects (e.g., environmental or technical covariates), which reduces the residual variance and increases the proportion of phenotypic variance attributable to additive genetic factors. Even when trying to account for sample size constraints using the model 2 approach, later analyses confirmed that the identified associated regions (Table 2 and 3) were the same irrespective of sample sizes. The outcomes of this study provide insight into the relationships of body attributes in a complex (UBTs) or simple manner (representative traits) that likely link back to common genomic regions. However, further validation and comparison of results with a much larger sample size is needed.

Considering the role of DENS and other traits, these identified regions were indicative of the body size-related nature of the data, as the regions were found to be associated with morphogenesis, energy metabolism, and growth-related traits (Saito et al., 2012; Feng et al., 2020; Smith et al., 2022). Differences in the identification of truly associated regions, as determined by the PPA_W_ thresholds, were influenced by the genetic relationships among the traits, their heritability, and the specific GWA methodology utilized (Fernando et al., 2017). Functional enrichment analysis using g:Profiler identified genes and GO terms involved in the Kyoto Encyclopedia of Genes and Genomics (KEGG; Kanehisa and Goto, 2000) signaling molecules and the interaction pathway of extracellular matrix (ECM)–receptor interaction (ID 04512). Protein coding genes in the associated regions in the ECM–receptor interaction pathway were *integrin-binding sialoprotein* (*IBSP*), *matrix extracellular phosphoglycoprotein* (*MEPE*), and *osteopontin phosphoprotein 1* (*SPP1*). All three of these genes are in close proximity on BTA 6 and play a role in regulating ECM cytoadhesion, which is directly associated to cellular growth, maintenance, and apoptosis and linked to milk, bone, and carcass characteristics (e.g., Alam et al., 2023; Chen et al., 2024; Wang et al., 2024). Aligning with this KEGG pathway were molecular functions and cellular components involving calcium channel activity and cellular structures. Considering other studies, the features found in this study have been identified playing similar roles for body size, reproductive characteristics, carcass, and growth. For example, there were enriched chromosomal regulatory pathways, including three genes from the *HERC* (*HECT and RLD Domain Containing E3 Ubiquitin Protein Ligase*) family, namely, *HERC3*, *HERC6*, and *HERC5*, which were involved in cellular growth and immunity related traits (Sánchez-Tena et al., 2016). Some desmosome protein associated adhesion pathways were found involving *desmocollin* genes (*DSC2* and *DSC3*) that are reported to be involved in morphogenesis (Saito et al., 2012), such as DENS, BS, and BC in this study. Similar to the *HERC* family, another ubiquitin–ligase complex pathway gene, *DCAF16*, was identified on BTA 6 (Table 3). Genes such as *FAM184B*, *NCAPG*, and *LCORL* were also found to be associated with economically important traits in cattle, including the average daily gain, body size, muscular development, and carcass traits (Takasuga, 2016; Zhang et al., 2016; Han et al., 2017; Smith et al., 2022). Energy metabolism-related pathways (phosphatidylinositol metabolic process and glycerophospholipid metabolic process) were found enriched for *PIGY* involved in carcass- and growth-related traits (Feng et al., 2020; Smith et al., 2022). Developmental pathway-related genes from BTA 20 (*STC2*), were involved in vascular development (Wat and Wat, 2014) and cellular metabolism (Gonzalez-Recio et al., 2018), respectively. Some other genes such as *NAP1L5*, *BARHL2*, and *ZNF644* were found associated with methylation pattern regulation (Zaitoun and Khatib, 2006; Bian et al., 2015; Ibeagha-Awemu et al., 2021). Other features found to be associated with phenotypes used in this study, when explored in literature, such as tRNAs (TRNAC-GCA and TRNAG-CCC), were found to be associated with growth and immunity-related traits (Seabury et al., 2017; Silva et al., 2020), while miRNA (bta-mir-2285b-2) was found to be associated with backfat (Sun et al., 2014). Given these known functions and features from the associated windows, we can summarize that the regions were relevantly associated with our developed UBT phenotypes and direct individual traits despite the smaller data sample sizes available in this study.

## 5 Conclusion

This study identified nine consistent genomic windows that house relevant genomic features across developed UBTs, independent traits, and varying levels of π using BayesB approaches with univariate and multivariate models. Sample size provided constraints on repeatability per analysis, but across analyses, there were commonalities, especially when considering the body size and density. This was evident when SEM approaches were used in model 1, which had the lowest sample size, but a stronger causal structure was estimated, indicating a more important relationship of carcass traits with body size that was lost in model 2’s approach. Furthermore, the DENS phenotype, which did not fit into a UBT, provided consistent alignment across other phenotypes and genomic regions that were identified, regardless of how those other phenotypes were analyzed. As DENS is the ratio of the body weight to volume, where volume uses body measures such as the girth and width, it is likely a similar UBT, but it is calculated from the direct metric equation rather than using factor analysis approaches to estimate how they are related. This study provides insights into the relationships of body- and carcass-oriented traits in admixed beef heifers that are directly relevant to the phenotyping and genetic evaluations being conducted in the beef industry.

## Data Availability

The datasets presented in this article are not readily available because of on-going research efforts. Requests to access the datasets should be directed to the corresponding author.

## Supplementary material

SUPPLEMENTARY FIGURE S1 Scripts for the genome-wide association analysis of all traits and modeling approaches following the BayesB approach in Julia along with the post-processing scripts in R.

SUPPLEMENTARY FIGURE S2 Combined MCMC and diagnostic summaries across the analyses.

SUPPLEMENTARY FIGURE S3 Combined posterior GWAA summaries across the analyses.

SUPPLEMENTARY FIGURE S4 g:Profiler enrichment analysis summary of features mapped to the nearby regions of the associated windows.
