# Supplementary material for: Genome-wide association study of the reproductive, body size, and carcass-related latent and directly measured traits in admixed beef heifers
Source: Front Genet. 2026 Jul 7;17:1653878. doi: 10.3389/fgene.2026.1653878 (PMC13384437; doi:10.3389/fgene.2026.1653878)
Supplement: Supplementary file 2 [file DataSheet1.pdf]

```
## Supplementary S1: JWAS script for genome-wide association analysis of developed underlying biological
## traits following BayesB approach and R script for processing of MCMC and GWAS output.

############# JULIA SCRIPT #############
#If you do not have packages called below installed already, then remove the hash and add them here:
#using Pkg
#Pkg.add("JWAS") #or others if needed

## Step 1: Load Packages
using JWAS,DataFrames,CSV,MCMCChains

########################################
####### UNIVARIATE ONLY ANALYSES #######
########################################

########################################
#### MODEL_1 UBT Body Size
#Set directory for data files...
cd("Path to folder with data files...")

## Step 2: Read Data
phenotypes = CSV.read("model1_pheno.txt",DataFrame,delim = '\t',header=true,missingstring=["NA"]);
pedigree = get_pedigree("pedigree2.txt",separator=",",header=true);
geno="geno_aligned_model1"
genotypes = get_genotypes(geno,method="BayesB",Pi=0.90,estimatePi=false,header=true);
#Pi = 0.001, Pi = 0.95 also used

## Step 3: Build Model Equations
model_equation = "Body_Size = intercept + Year + DA + PBG + ID + genotypes";
model=build_model(model_equation)

## Step 4: Set Factors or Covariates
## None needed

## Step 5: Set Random or Fixed Effects
set_random(model,"ID",pedigree);

## Step 6: Run Bayesian Analysis
out=runMCMC(model,phenotypes,chain_length=210000,burnin=10000,output_samples_frequency=100);

##Diagnostics and summary in one place (redone in R script)
sigma_a = Array(CSV.read("results/MCMC_samples_genetic_variance.txt",DataFrame,delim = ' ',header=true));
sigma_e = Array(CSV.read("results/MCMC_samples_residual_variance.txt",DataFrame,delim = ' ',header=true));
h2 = Array(CSV.read("results/MCMC_samples_heritability.txt",DataFrame,delim = ' ',header=true));
samples = Chains(hcat(sigma_a,sigma_e,h2),[:sigma_a,:sigma_e,:h2])

path = joinpath(pwd(),"results/MCMC_summary.txt")
open(path, "w") do io
    describe(io,samples)
end

#Copy map file so it is available in the new directory...
cp("Map_Ordered.csv","results/Map_Ordered.csv")
#Set directory GWAS files...
cd("Path to folder with data files.../results")

## Step 7: Genome-Wide Association Analysis
map_file="Map_Ordered.csv";
marker_effects_file1 ="MCMC_samples_marker_effects_genotypes_Body_Size.txt"
out=GWAS(model,map_file,marker_effects_file1,header=true,window_size="1 Mb");

########################################
```

```
#### MODEL_1 UBT Body Composition
#Set directory for data files...
cd("Path to folder with data files...")
mv("results","results_BS1_BB") #rename folder of last run to keep from overwriting

## Step 2: Read Data
phenotypes = CSV.read("model1_pheno.txt",DataFrame,delim = '\t',header=true,missingstring=["NA"]);
pedigree = get_pedigree("pedigree2.txt",separator=",",header=true);
geno="geno_aligned_model1"
genotypes = get_genotypes(geno,method="BayesB",Pi=0.90,estimatePi=false,header=true);
#Pi = 0.001, Pi = 0.95 also used

## Step 3: Build Model Equations
model_equation = "Body_Composition = intercept + Year + DA + PBG + ID + genotypes";
model=build_model(model_equation)

## Step 4: Set Factors or Covariates
## None needed

## Step 5: Set Random or Fixed Effects
set_random(model,"ID",pedigree);

## Step 6: Run Bayesian Analysis
out=runMCMC(model,phenotypes,chain_length=210000,burnin=10000,output_samples_frequency=100);

##Diagnostics and summary in one place (redone in R script)
sigma_a = Array(CSV.read("results/MCMC_samples_genetic_variance.txt",DataFrame,delim = ' ',header=true));
sigma_e = Array(CSV.read("results/MCMC_samples_residual_variance.txt",DataFrame,delim = ' ',header=true));
h2 = Array(CSV.read("results/MCMC_samples_heritability.txt",DataFrame,delim = ' ',header=true));
samples = Chains(hcat(sigma_a,sigma_e,h2),[:sigma_a,:sigma_e,:h2])

path = joinpath(pwd(),"results/MCMC_summary.txt")
open(path, "w") do io
    describe(io,samples)
end

#Copy map file so it is available in the new directory...
cp("Map_Ordered.csv","results/Map_Ordered.csv")
#Set directory GWAS files...
cd("Path to folder with data files.../results")

## Step 7: Genome-Wide Association Analysis
map_file="Map_Ordered.csv";
marker_effects_file1 ="MCMC_samples_marker_effects_genotypes_Body_Composition.txt"
out=GWAS(model,map_file,marker_effects_file1,header=true,window_size="1 Mb");

########################################
#### MODEL_2 UBT Body Size
#Set directory for data files...
cd("Path to folder with data files...")
mv("results","results_BC_BB") #rename folder to keep from overwriting

## Step 2: Read Data
phenotypes = CSV.read("model2_body_pheno.txt",DataFrame,delim = '\t',header=true,missingstring=["NA"]);
pedigree = get_pedigree("pedigree2.txt",separator=",",header=true);
geno="geno_aligned_body_model2"
genotypes = get_genotypes(geno,method="BayesB",Pi=0.90,estimatePi=false,header=true);
#Pi = 0.001, Pi = 0.95 also used

## Step 3: Build Model Equations
model_equation = "Body_Size = intercept + Year + DA + PBG + ID + genotypes";
model=build_model(model_equation)
```

```
## Step 4: Set Factors or Covariates
## None needed

## Step 5: Set Random or Fixed Effects
set_random(model,"ID",pedigree);

## Step 6: Run Bayesian Analysis
out=runMCMC(model,phenotypes,chain_length=210000,burnin=10000,output_samples_frequency=100);

##Diagnostics and summary in one place (redone in R script)
sigma_a = Array(CSV.read("results/MCMC_samples_genetic_variance.txt",DataFrame,delim = ' ',header=true));
sigma_e = Array(CSV.read("results/MCMC_samples_residual_variance.txt",DataFrame,delim = ' ',header=true));
h2 = Array(CSV.read("results/MCMC_samples_heritability.txt",DataFrame,delim = ' ',header=true));
samples = Chains(hcat(sigma_a,sigma_e,h2),[:sigma_a,:sigma_e,:h2])

path = joinpath(pwd(),"results/MCMC_summary.txt")
open(path, "w") do io
    describe(io,samples)
end

#Copy map file so it is available in the new directory...
cp("Map_Ordered.csv","results/Map_Ordered.csv")
#Set directory GWAS files...
cd("Path to folder with data files.../results")

## Step 7: Genome-Wide Association Analysis
map_file="Map_Ordered.csv";
marker_effects_file1 ="MCMC_samples_marker_effects_genotypes_Body_Size.txt"
out=GWAS(model,map_file,marker_effects_file1,header=true,window_size="1 Mb");

########################################
#### MODEL_2 UBT Ovary Size
#Set directory for data files...
cd("Path to folder with data files...")
mv("results","results_BS2_BB") #rename folder to keep from overwriting

## Step 2: Read Data
phenotypes = CSV.read("model2_body_pheno.txt",DataFrame,delim = '\t',header=true,missingstring=["NA"]);
pedigree = get_pedigree("pedigree2.txt",separator=",",header=true);
geno="geno_aligned_body_model2"
genotypes = get_genotypes(geno,method="BayesB",Pi=0.90,estimatePi=false,header=true);
#Pi = 0.001, Pi = 0.95 also used

## Step 3: Build Model Equations
model_equation = "Ovary_Size = intercept + Year + DA + PBG + ID + genotypes";
model=build_model(model_equation)

## Step 4: Set Factors or Covariates
## None needed

## Step 5: Set Random or Fixed Effects
set_random(model,"ID",pedigree);

## Step 6: Run Bayesian Analysis
out=runMCMC(model,phenotypes,chain_length=210000,burnin=10000,output_samples_frequency=100);

##Diagnostics and summary in one place (redone in R script)
sigma_a = Array(CSV.read("results/MCMC_samples_genetic_variance.txt",DataFrame,delim = ' ',header=true));
sigma_e = Array(CSV.read("results/MCMC_samples_residual_variance.txt",DataFrame,delim = ' ',header=true));
h2 = Array(CSV.read("results/MCMC_samples_heritability.txt",DataFrame,delim = ' ',header=true));
```

```
samples = Chains(hcat(sigma_a,sigma_e,h2),[:sigma_a,:sigma_e,:h2])

path = joinpath(pwd(),"results/MCMC_summary.txt")
open(path, "w") do io
    describe(io,samples)
end

#Copy map file so it is available in the new directory...
cp("Map_Ordered.csv","results/Map_Ordered.csv")
#Set directory GWAS files...
cd("Path to folder with data files.../results")

## Step 7: Genome-Wide Association Analysis
map_file="Map_Ordered.csv";
marker_effects_file1 ="MCMC_samples_marker_effects_genotypes_Ovary_Size.txt"
out=GWAS(model,map_file,marker_effects_file1,header=true,window_size="1 Mb");

########################################
#### MODEL_2 UBT Yield Grade
#Set directory for data files...
cd("Path to folder with data files...")
mv("results","results_OS_BB") #rename folder to keep from overwriting

## Step 2: Read Data
phenotypes = CSV.read("model2_carcass_pheno.txt",DataFrame,delim = '\t',header=true,missingstring=["NA"]);
pedigree = get_pedigree("pedigree2.txt",separator=",",header=true);
geno="geno_aligned_carcass_model2"
genotypes = get_genotypes(geno,method="BayesB",Pi=0.90,estimatePi=false,header=true);
#Pi = 0.001, Pi = 0.95 also used

## Step 3: Build Model Equations
model_equation = "Yield_Grade = intercept + Year + DA + PBG + ID + genotypes";
model = build_model(model_equation);

## Step 4: Set Factors or Covariates
## None needed

## Step 5: Set Random or Fixed Effects
set_random(model,"ID",pedigree);

## Step 6: Run Bayesian Analysis
out=runMCMC(model,phenotypes,chain_length=210000,burnin=10000,output_samples_frequency=100);

##Diagnostics and summary in one place (redone in R script)
sigma_a = Array(CSV.read("results/MCMC_samples_genetic_variance.txt",DataFrame,delim = ' ',header=true));
sigma_e = Array(CSV.read("results/MCMC_samples_residual_variance.txt",DataFrame,delim = ' ',header=true));
h2 = Array(CSV.read("results/MCMC_samples_heritability.txt",DataFrame,delim = ' ',header=true));
samples = Chains(hcat(sigma_a,sigma_e,h2),[:sigma_a,:sigma_e,:h2])

path = joinpath(pwd(),"results/MCMC_summary.txt")
open(path, "w") do io
    describe(io,samples)
end

#Copy map file so it is available in the new directory...
cp("Map_Ordered.csv","results/Map_Ordered.csv")
#Set directory GWAS files...
cd("Path to folder with data files.../results")

## Step 7: Genome-Wide Association Analysis
map_file="Map_Ordered.csv";
marker_effects_file1 ="MCMC_samples_marker_effects_genotypes_Yield_Grade.txt"
out=GWAS(model,map_file,marker_effects_file1,header=true,window_size="1 Mb");
```

```
########################################
#### NC-ERT Intramuscular Fat
#Set directory for data files...
cd("Path to folder with data files...")
mv("results","results_YG_BB") #rename folder to keep from overwriting

## Step 2: Read Data
phenotypes = CSV.read("model2_carcass_pheno.txt",DataFrame,delim = '\t',header=true,missingstring=["NA"]);
pedigree = get_pedigree("pedigree2.txt",separator=",",header=true);
geno="geno_aligned_carcass_model2"
genotypes = get_genotypes(geno,method="BayesB",Pi=0.90,estimatePi=false,header=true);
#Pi = 0.001, Pi = 0.95 also used

## Step 3: Build Model Equations
model_equation = "iIMF = intercept + Year + DA + PBG + ID + genotypes";
model = build_model(model_equation);

## Step 4: Set Factors or Covariates
## None needed

## Step 5: Set Random or Fixed Effects
set_random(model,"ID",pedigree);

## Step 6: Run Bayesian Analysis
out=runMCMC(model,phenotypes,chain_length=210000,burnin=10000,output_samples_frequency=100);

##Diagnostics and summary in one place (redone in R script)
sigma_a = Array(CSV.read("results/MCMC_samples_genetic_variance.txt",DataFrame,delim = ' ',header=true));
sigma_e = Array(CSV.read("results/MCMC_samples_residual_variance.txt",DataFrame,delim = ' ',header=true));
h2 = Array(CSV.read("results/MCMC_samples_heritability.txt",DataFrame,delim = ' ',header=true));
samples = Chains(hcat(sigma_a,sigma_e,h2),[:sigma_a,:sigma_e,:h2])

path = joinpath(pwd(),"results/MCMC_summary.txt")
open(path, "w") do io
    describe(io,samples)
end

#Copy map file so it is available in the new directory...
cp("Map_Ordered.csv","results/Map_Ordered.csv")
#Set directory GWAS files...
cd("Path to folder with data files.../results")

## Step 7: Genome-Wide Association Analysis
map_file="Map_Ordered.csv";
marker_effects_file1 ="MCMC_samples_marker_effects_genotypes_iIMF.txt"
out=GWAS(model,map_file,marker_effects_file1,header=true,window_size="1 Mb");

########################################
#### NC-ERT Density
#Set directory for data files...
cd("Path to folder with data files...")
mv("results","results_iIMF_BB") #rename folder to keep from overwriting

## Step 2: Read Data
phenotypes = CSV.read("model2_body_pheno.txt",DataFrame,delim = '\t',header=true,missingstring=["NA"]);
pedigree = get_pedigree("pedigree2.txt",separator=",",header=true);
geno="geno_aligned_body_model2"
genotypes = get_genotypes(geno,method="BayesB",Pi=0.90,estimatePi=false,header=true);
#Pi = 0.001, Pi = 0.95 also used

## Step 3: Build Model Equations
model_equation = "iDENS = intercept + Year + DA + PBG + ID + genotypes";
model = build_model(model_equation);
```

```
## Step 4: Set Factors or Covariates
## None needed

## Step 5: Set Random or Fixed Effects
set_random(model,"ID",pedigree);

## Step 6: Run Bayesian Analysis
out=runMCMC(model,phenotypes,chain_length=210000,burnin=10000,output_samples_frequency=100);

##Diagnostics and summary in one place (redone in R script)
sigma_a = Array(CSV.read("results/MCMC_samples_genetic_variance.txt",DataFrame,delim = ' ',header=true));
sigma_e = Array(CSV.read("results/MCMC_samples_residual_variance.txt",DataFrame,delim = ' ',header=true));
h2 = Array(CSV.read("results/MCMC_samples_heritability.txt",DataFrame,delim = ' ',header=true));
samples = Chains(hcat(sigma_a,sigma_e,h2),[:sigma_a,:sigma_e,:h2])

path = joinpath(pwd(),"results/MCMC_summary.txt")
open(path, "w") do io
    describe(io,samples)
end

#Copy map file so it is available in the new directory...
cp("Map_Ordered.csv","results/Map_Ordered.csv")
#Set directory GWAS files...
cd("Path to folder with data files.../results")

## Step 7: Genome-Wide Association Analysis
map_file="Map_Ordered.csv";
marker_effects_file1 ="MCMC_samples_marker_effects_genotypes_iDENS.txt"
out=GWAS(model,map_file,marker_effects_file1,header=true,window_size="1 Mb");

########################################
#### REP. TRAIT Body Weight
#Set directory for data files...
cd("Path to folder with data files...")
mv("results","results_iDENS_BB") #rename folder to keep from overwriting

## Step 2: Read Data
phenotypes = CSV.read("model2_body_pheno.txt",DataFrame,delim = '\t',header=true,missingstring=["NA"]);
pedigree = get_pedigree("pedigree2.txt",separator=",",header=true);
geno="geno_aligned_body_model2"
genotypes = get_genotypes(geno,method="BayesB",Pi=0.90,estimatePi=false,header=true);
#Pi = 0.001, Pi = 0.95 also used

## Step 3: Build Model Equations
model_equation = "iBWT = intercept + Year + DA + PBG + ID + genotypes";
model = build_model(model_equation);

## Step 4: Set Factors or Covariates
## None needed

## Step 5: Set Random or Fixed Effects
set_random(model,"ID",pedigree);

## Step 6: Run Bayesian Analysis
out=runMCMC(model,phenotypes,chain_length=210000,burnin=10000,output_samples_frequency=100);

##Diagnostics and summary in one place (redone in R script)
sigma_a = Array(CSV.read("results/MCMC_samples_genetic_variance.txt",DataFrame,delim = ' ',header=true));
sigma_e = Array(CSV.read("results/MCMC_samples_residual_variance.txt",DataFrame,delim = ' ',header=true));
h2 = Array(CSV.read("results/MCMC_samples_heritability.txt",DataFrame,delim = '
```

```
',header=true));
samples = Chains(hcat(sigma_a,sigma_e,h2),[:sigma_a,:sigma_e,:h2])

path = joinpath(pwd(),"results/MCMC_summary.txt")
open(path, "w") do io
    describe(io,samples)
end

#Copy map file so it is available in the new directory...
cp("Map_Ordered.csv","results/Map_Ordered.csv")
#Set directory GWAS files...
cd("Path to folder with data files.../results")

## Step 7: Genome-Wide Association Analysis
map_file="Map_Ordered.csv";
marker_effects_file1 ="MCMC_samples_marker_effects_genotypes_iBWT.txt"
out=GWAS(model,map_file,marker_effects_file1,header=true,window_size="1 Mb");

########################################
#### REP. TRAIT Antral Follicle Count
#Set directory for data files...
cd("Path to folder with data files...")
mv("results","results_iBWT_BB") #rename folder to keep from overwriting

## Step 2: Read Data
phenotypes = CSV.read("model2_body_pheno.txt",DataFrame,delim =
'\t',header=true,missingstring=["NA"]);
pedigree = get_pedigree("pedigree2.txt",separator=",",header=true);
geno="geno_aligned_body_model2"
genotypes = get_genotypes(geno,method="BayesB",Pi=0.90,estimatePi=false,header=true);
#Pi = 0.001, Pi = 0.95 also used

## Step 3: Build Model Equations
model_equation = "AFC = intercept + Year + DA + PBG + ID + genotypes";
model = build_model(model_equation);

## Step 4: Set Factors or Covariates
## None needed

## Step 5: Set Random or Fixed Effects
set_random(model,"ID",pedigree);

## Step 6: Run Bayesian Analysis
out=runMCMC(model,phenotypes,chain_length=210000,burnin=10000,output_samples_frequency=10
0);

##Diagnostics and summary in one place (redone in R script)
sigma_a = Array(CSV.read("results/MCMC_samples_genetic_variance.txt",DataFrame,delim = '
',header=true));
sigma_e = Array(CSV.read("results/MCMC_samples_residual_variance.txt",DataFrame,delim =
' ',header=true));
h2 = Array(CSV.read("results/MCMC_samples_heritability.txt",DataFrame,delim = '
',header=true));
samples = Chains(hcat(sigma_a,sigma_e,h2),[:sigma_a,:sigma_e,:h2])

path = joinpath(pwd(),"results/MCMC_summary.txt")
open(path, "w") do io
    describe(io,samples)
end

#Copy map file so it is available in the new directory...
cp("Map_Ordered.csv","results/Map_Ordered.csv")
#Set directory GWAS files...
cd("Path to folder with data files.../results")

## Step 7: Genome-Wide Association Analysis
map_file="Map_Ordered.csv";
marker_effects_file1 ="MCMC_samples_marker_effects_genotypes_AFC.txt"
out=GWAS(model,map_file,marker_effects_file1,header=true,window_size="1 Mb");
```

```
########################################
#### REP. TRAIT Yield Grade
#Set directory for data files...
cd("Path to folder with data files...")
mv("results","results_AFC_BB") #rename folder to keep from overwriting

## Step 2: Read Data
phenotypes = CSV.read("model2_carcass_pheno.txt",DataFrame,delim =
'\t',header=true,missingstring=["NA"]);
pedigree = get_pedigree("pedigree2.txt",separator=",",header=true);
geno="geno_aligned_carcass_model2"
genotypes = get_genotypes(geno,method="BayesB",Pi=0.90,estimatePi=false,header=true);
#Pi = 0.001, Pi = 0.95 also used

## Step 3: Build Model Equations
model_equation = "iYG = intercept + Year + DA + PBG + ID + genotypes";
model = build_model(model_equation);

## Step 4: Set Factors or Covariates
## None needed

## Step 5: Set Random or Fixed Effects
set_random(model,"ID",pedigree);

## Step 6: Run Bayesian Analysis
out=runMCMC(model,phenotypes,chain_length=210000,burnin=10000,output_samples_frequency=10
0);

##Diagnostics and summary in one place (redone in R script)
sigma_a = Array(CSV.read("results/MCMC_samples_genetic_variance.txt",DataFrame,delim = '
',header=true));
sigma_e = Array(CSV.read("results/MCMC_samples_residual_variance.txt",DataFrame,delim =
' ',header=true));
h2 = Array(CSV.read("results/MCMC_samples_heritability.txt",DataFrame,delim = '
',header=true));
samples = Chains(hcat(sigma_a,sigma_e,h2),[:sigma_a,:sigma_e,:h2])

path = joinpath(pwd(),"results/MCMC_summary.txt")
open(path, "w") do io
    describe(io,samples)
end

#Copy map file so it is available in the new directory...
cp("Map_Ordered.csv","results/Map_Ordered.csv")
#Set directory GWAS files...
cd("Path to folder with data files.../results")

## Step 7: Genome-Wide Association Analysis
map_file="Map_Ordered.csv";
marker_effects_file1 ="MCMC_samples_marker_effects_genotypes_iYG.txt"
out=GWAS(model,map_file,marker_effects_file1,header=true,window_size="1 Mb");

################################################################
####### MULTIVARIATE ANALYSES - NO CORRELATION STRUCTURE #######
################################################################

########################################
#### MODEL_1: Multi-Trait GWAS for Body_Size and Body_Composition
#Set directory for data files...
cd("Path to folder with data files...")
mv("results","results_iYG_BB") #rename folder to keep from overwriting

## Step 2: Read Data
phenotypes = CSV.read("model1_pheno.txt",DataFrame,delim =
'\t',header=true,missingstring=["NA"]);
pedigree = get_pedigree("pedigree2.txt",separator=",",header=true);
geno="geno_aligned_model1"
myPi = Dict([1.0;1.0]=>0.05,[1.0;0.0]=>0.025,[0.0;1.0]=>0.025,[0.0;0.0]=>0.90)
```

```
#myPi = Dict([1.0;1.0]=>0.002,[1.0;0.0]=>0.001,[0.0;1.0]=>0.001,[0.0;0.0]=>0.996) as another option, but too sparse
genotypes = get_genotypes(geno,method="BayesB",Pi=myPi,estimatePi=false,header=true);

## Step 3: Build Model Equations
model_equation = "Body_Size = intercept + Year + DA + PBG + ID + genotypes
                  Body_Composition= intercept + Year + DA + PBG + ID + genotypes";
model = build_model(model_equation);

## Step 4: Set Factors or Covariates
## None needed

## Step 5: Set Random or Fixed Effects
set_random(model,"ID",pedigree);

## Step 6: Run Bayesian Analysis
out=runMCMC(model,phenotypes,chain_length=210000,burnin=10000,output_samples_frequency=100);

##Diagnostics and summary in one place (redone in R script)
sigma_a = CSV.read("results/MCMC_samples_genetic_variance.txt",DataFrame,delim = ',',header=true);
rename!(n -> n*"_sa",sigma_a)

sigma_e = CSV.read("results/MCMC_samples_residual_variance.txt",DataFrame,delim = ',',header=true);
rename!(n -> n*"_se",sigma_e)

h2 = CSV.read("results/MCMC_samples_heritability.txt",DataFrame,delim = ',',header=true);
rename!(n -> n*"_h2",h2)

colnames = vcat(names(sigma_a),names(sigma_e),names(h2))
samples = Chains(hcat(Array(sigma_a),Array(sigma_e),Array(h2)),colnames);

path = joinpath(pwd(),"results/MCMC_summary.txt");
open(path, "w") do io
    describe(io,samples)
end

#Copy map file so it is available in the new directory...
cp("Map_Ordered.csv","results/Map_Ordered.csv")
#Set directory GWAS files...
cd("Path to folder with data files.../results")

## Step 7: Genome-Wide Association Analysis
map_file="Map_Ordered.csv";
marker_effects_file1 ="MCMC_samples_marker_effects_genotypes_Body_Size.txt"
marker_effects_file2 ="MCMC_samples_marker_effects_genotypes_Body_Composition.txt"
out=GWAS(model,map_file,marker_effects_file1,marker_effects_file2,header=true,window_size="1 Mb");

#######################################
#### MODEL_2: Multi-Trait for Ovary_Size and Body_Size
#Set directory for data files...
cd("Path to folder with data files...")
mv("results","results_MTM1_BB") #rename folder to keep from overwriting

## Step 2: Read Data
phenotypes = CSV.read("model2_body_pheno.txt",DataFrame,delim = '\t',header=true,missingstring=["NA"]);
pedigree = get_pedigree("pedigree2.txt",separator=",",header=true);
geno="geno_aligned_body_model2"
myPi = Dict([1.0;1.0]=>0.05,[1.0;0.0]=>0.025,[0.0;1.0]=>0.025,[0.0;0.0]=>0.90)
#myPi = Dict([1.0;1.0]=>0.002,[1.0;0.0]=>0.001,[0.0;1.0]=>0.001,[0.0;0.0]=>0.996) as another option, but too sparse
genotypes = get_genotypes(geno,method="BayesB",Pi=myPi,estimatePi=false,header=true);

## Step 3: Build Model Equations
model_equation = "Body_Size = intercept + Year + DA + PBG + ID + genotypes
```

```
                     Ovary_Size = intercept + Year + DA + PBG + ID + genotypes";
model = build_model(model_equation);

## Step 4: Set Factors or Covariates
## None needed

## Step 5: Set Random or Fixed Effects
set_random(model,"ID",pedigree);

## Step 6: Run Bayesian Analysis
out=runMCMC(model,phenotypes,chain_length=210000,burnin=10000,output_samples_frequency=100);

##Diagnostics and summary in one place (redone in R script)
sigma_a = CSV.read("results/MCMC_samples_genetic_variance.txt",DataFrame,delim = ',',header=true);
rename!(n -> n*"_sa",sigma_a)

sigma_e = CSV.read("results/MCMC_samples_residual_variance.txt",DataFrame,delim = ',',header=true);
rename!(n -> n*"_se",sigma_e)

h2 = CSV.read("results/MCMC_samples_heritability.txt",DataFrame,delim = ',',header=true);
rename!(n -> n*"_h2",h2)

colnames = vcat(names(sigma_a),names(sigma_e),names(h2))
samples = Chains(hcat(Array(sigma_a),Array(sigma_e),Array(h2)),colnames);

path = joinpath(pwd(),"results/MCMC_summary.txt")
open(path, "w") do io
    describe(io,samples)
end

#Copy map file so it is available in the new directory...
cp("Map_Ordered.csv","results/Map_Ordered.csv")
#Set directory GWAS files...
cd("Path to folder with data files.../results")

## Step 7: Genome-Wide Association Analysis
map_file="Map_Ordered.csv";
marker_effects_file1 ="MCMC_samples_marker_effects_genotypes_Body_Size.txt"
marker_effects_file2 ="MCMC_samples_marker_effects_genotypes_Ovary_Size.txt"
out=GWAS(model,map_file,marker_effects_file1,marker_effects_file2,header=true,window_size="1 Mb");

#########################################################
####### SEM ANALYSES - CORRELATION STRUCTURE USED #######
#########################################################

#########################################
#### MODEL_1: Body Size Influencing Body Composition
#Set directory for data files...
cd("Path to folder with data files...")
mv("results","results_MTM2_BB") #rename folder to keep from overwriting

## Step 2: Read Data
phenotypes = CSV.read("model1_pheno.txt",DataFrame,delim = '\t',header=true,missingstring=["NA"]);
pedigree = get_pedigree("pedigree2.txt",separator=",",header=true);
geno="geno_aligned_model1"
myPi = Dict([1.0;1.0]=>0.05,[1.0;0.0]=>0.025,[0.0;1.0]=>0.025,[0.0;0.0]=>0.90)
#myPi = Dict([1.0;1.0]=>0.002,[1.0;0.0]=>0.001,[0.0;1.0]=>0.001,[0.0;0.0]=>0.996) as another option, but too sparse
genotypes = get_genotypes(geno,method="BayesB",Pi=myPi,estimatePi=false,header=true);

## Step 3: Build Model Equations
model_equation = "Body_Size = intercept + Year + DA + PBG + ID + genotypes
                     Body_Composition= intercept + Year + DA + PBG + ID + genotypes";
model = build_model(model_equation);
```

```
## Step 4: Set Factors or Covariates
## None needed

## Step 5: Set Random or Fixed Effects
set_random(model,"ID",pedigree);

## Step 6: Run Bayesian Analysis
#Including the Bayesian Structure for SEM
my_structure = [0.0 0.0
                1.0 0.0]
out=runMCMC(model,phenotypes,causal_structure=my_structure,chain_length=210000,burnin=10000,output_samples_frequency=100);
cp("structure_coefficient_MCMC_samples.txt","results/structure_coefficient_MCMC_samples.txt")

##Diagnostics and summary in one place (redone in R script)
sigma_a = CSV.read("results/MCMC_samples_genetic_variance.txt",DataFrame,delim = ',',header=true);
rename!(n -> n*"_sa",sigma_a)

sigma_e = CSV.read("results/MCMC_samples_residual_variance.txt",DataFrame,delim = ',',header=true);
rename!(n -> n*"_se",sigma_e)

h2 = CSV.read("results/MCMC_samples_heritability.txt",DataFrame,delim = ',',header=true);
rename!(n -> n*"_h2",h2)

castruct = CSV.read("results/structure_coefficient_MCMC_samples.txt",DataFrame,delim = ',',header=false);

colnames = vcat(names(sigma_a),names(sigma_e),names(h2),"ca11","ca12","ca21","ca22")
samples = Chains(hcat(Array(sigma_a),Array(sigma_e),Array(h2),Array(castruct)),colnames);

path = joinpath(pwd(),"results/MCMC_summary.txt")
open(path, "w") do io
    describe(io,samples)
end

#Copy map file so it is available in the new directory...
cp("Map_Ordered.csv","results/Map_Ordered.csv")
#Set directory GWAS files...
cd("Path to folder with data files.../results")

## Step 7: Genome-Wide Association Analysis
# Step 7.1: GWAS, Direct Marker Effects on Trait Body Composition
map_file="Map_Ordered.csv";
marker_effects_file="MCMC_samples_marker_effects_genotypes_Body_Composition.txt";
outs=GWAS(model,map_file,marker_effects_file,header=true,window_size="1 Mb");

# Step 7.2: GWAS, Indirect Marker Effects on Body Composition
marker_effects_file2="MCMC_samples_indirect_marker_effects_genotypes_Body_Composition.txt"
outs2=GWAS(model,map_file,marker_effects_file2,header=true,window_size="1 Mb");

# Step 7.3: GWAS, Overall Marker Effects on Trait Body Composition
marker_effects_file3="MCMC_samples_overall_marker_effects_genotypes_Body_Composition.txt"
outs3=GWAS(model,map_file,marker_effects_file3,header=true,window_size="1 Mb");

# Step 7.4: GWAS, Overall Marker Effects on Trait Body Size
marker_effects_file4="MCMC_samples_overall_marker_effects_genotypes_Body_Size.txt"
outs4=GWAS(model,map_file,marker_effects_file4,header=true,window_size="1 Mb");

########################################
#### MODEL_2: Body Size influencing Ovary Size
#Set directory for data files...
cd("Path to folder with data files...")
mv("results","results_SEM1_BB") #rename folder to keep from overwriting
```

```
## Step 2: Read Data
phenotypes = CSV.read("model2_body_pheno.txt",DataFrame,delim = '\t',header=true,missingstring=["NA"]);
pedigree = get_pedigree("pedigree2.txt",separator=",",header=true);
geno="geno_aligned_body_model2"
myPi = Dict([1.0;1.0]=>0.05,[1.0;0.0]=>0.025,[0.0;1.0]=>0.025,[0.0;0.0]=>0.90)
#myPi = Dict([1.0;1.0]=>0.002,[1.0;0.0]=>0.001,[0.0;1.0]=>0.001,[0.0;0.0]=>0.996) as another option, but too sparse
genotypes = get_genotypes(geno,method="BayesB",Pi=myPi,estimatePi=false,header=true);

## Step 3: Build Model Equations
model_equation = "Body_Size = intercept + Year + DA + PBG + ID + genotypes
                  Ovary_Size= intercept + Year + DA + PBG + ID + genotypes";
model = build_model(model_equation);

## Step 4: Set Factors or Covariates
## None needed

## Step 5: Set Random or Fixed Effects
set_random(model,"ID",pedigree);

## Step 6: Run Bayesian Analysis
##Including the Bayesian Structure for SEM
my_structure = [0.0 0.0
                1.0 0.0];
out=runMCMC(model,phenotypes,causal_structure=my_structure,chain_length=210000,burnin=10000,output_samples_frequency=100);
cp("structure_coefficient_MCMC_samples.txt","results/structure_coefficient_MCMC_samples.txt")

##Diagnostics and summary in one place (redone in R script)
sigma_a = CSV.read("results/MCMC_samples_genetic_variance.txt",DataFrame,delim = ',',header=true);
rename!(n -> n*"_sa",sigma_a)

sigma_e = CSV.read("results/MCMC_samples_residual_variance.txt",DataFrame,delim = ',',header=true);
rename!(n -> n*"_se",sigma_e)

h2 = CSV.read("results/MCMC_samples_heritability.txt",DataFrame,delim = ',',header=true);
rename!(n -> n*"_h2",h2)

castruct = CSV.read("results/structure_coefficient_MCMC_samples.txt",DataFrame,delim = ',',header=false);

colnames = vcat(names(sigma_a),names(sigma_e),names(h2),"ca11","ca12","ca21","ca22")
samples = Chains(hcat(Array(sigma_a),Array(sigma_e),Array(h2),Array(castruct)),colnames);

path = joinpath(pwd(),"results/MCMC_summary.txt")
open(path, "w") do io
    describe(io,samples)
end

#Copy map file so it is available in the new directory...
cp("Map_Ordered.csv","results/Map_Ordered.csv")
#Set directory GWAS files...
cd("Path to folder with data files.../results")

## Step 7: Genome-Wide Association Analysis
# Step 7.1: GWAS, Direct Marker Effects on Trait Ovary_Size
#Compute the model frequency for each marker (the probability the marker is included in the model).
map_file="Map_Ordered.csv";
marker_effects_file="MCMC_samples_marker_effects_genotypes_Ovary_Size.txt";
outs=GWAS(model,map_file,marker_effects_file,header=true,window_size="1 Mb");

# Step 7.2: GWAS, Indirect Marker Effects on Ovary_Size
marker_effects_file2="MCMC_samples_indirect_marker_effects_genotypes_Ovary_Size.txt"
outs2=GWAS(model,map_file,marker_effects_file2,header=true,window_size="1 Mb");
```

```
# Step 7.3: GWAS, Overall Marker Effects on Trait Ovary_Size
marker_effects_file3="MCMC_samples_overall_marker_effects_genotypes_Ovary_Size.txt"
outs3=GWAS(model,map_file,marker_effects_file3,header=true,window_size="1 Mb");

# Step 7.4: GWAS, Overall Marker Effects on Trait Body Size
marker_effects_file4="MCMC_samples_overall_marker_effects_genotypes_Body_Size.txt"
outs4=GWAS(model,map_file,marker_effects_file4,header=true,window_size="1 Mb");

cd("Path to folder with data files...")
mv("results","results_SEM2_BB") #rename folder to keep from overwriting

############# R SCRIPT #############
##################################################
### Make sure an R file with script is placed in the folder with other folders of JWAS output
### Combine MCMC samples across multiple analyses into a single file:

#Find folders in current directory & extract traits involved
fold = list.dirs()
traits = array(gsub("./results_","",fold))
traits = array(gsub("_BB","",traits))

#Provide MCMC parameter sample options given analyses
MCMC.out = array(c("genetic_variance","residual_variance","heritability","structure_coefficient"))
Types = array(c("GenVar","ResidVar","Herit","CaSt"))

#Create base of iterations to align samples to; current study kept 2000 MCMC samples per analysis
samples = data.frame(Iteration = seq(1,2000,by=1))

#Process, per folder, files that have MCMC samples if relevance...
for(i in 1:dim(array(fold))){
  out = c(list.files(path = fold[i],pattern="^MCMC_samples"),list.files(path = fold[i],pattern="^structure_coefficient"))
  out = array(c(subset(out,grepl(MCMC.out[1],out)),subset(out,grepl(MCMC.out[2],out)),subset(out,grepl(MCMC.out[3],out)),subset(out,grepl(MCMC.out[4],out))))
  if(dim(array(out))==0){
    next
  }else{
    if(traits[i]=="MTM1" | traits[i]=="MTM2" |traits[i]=="SEM1" |traits[i]=="SEM2"){
      for(j in 1:dim(out)){
        if(j < 4){
          temp = read.table(paste0(fold[i],"/",out[j]),sep=",",header=TRUE)
          sub.traits = gsub("[^A-Z]","",colnames(temp))
          new.names = paste0(traits[i],"_",sub.traits,"_",Types[j])
        }else{ #causal structure does not have header
          temp = read.table(paste0(fold[i],"/",out[j]),sep=",",header=FALSE)
          temp = temp[,2,drop=FALSE]
          new.names = paste0(traits[i],"_ca12")
        }
        names(temp) = new.names
        if(j == 1){
          temp[,paste0(traits[i],"_GenCorr")] = temp[,2] / sqrt(temp[,1]*temp[,4])
        }
        samples = cbind(samples,temp)
      }
    }else{
        for(j in 1:dim(out)){
        temp = read.table(paste0(fold[i],"/",out[j]),sep=",",header=TRUE)
        new.names = paste0(traits[i],"_",Types[j])
        names(temp) = new.names
        samples = cbind(samples,temp)
      }
    }
  }
}
```

```
write.table(samples,"CombinedMCMCSamples.txt",sep=",",row.names=FALSE,quote=FALSE)
#clean up environment....
remove(temp,i,j,MCMC.out,new.names,out,fold)

#Given combined MCMC samples, conduct summary and diagnostic analyses, then store information
library(coda)
options(scipen = 999) #keep high threshold to prevent scientific notation...

sample.chains = mcmc(as.matrix(samples[,2:dim(samples)[2]]))
write.table(effectiveSize(sample.chains),"CombinedEffectiveSampleSize.txt",sep=",",row.names=TRUE,col.names=FALSE,quote=FALSE)

#Create summary groups & output
GenVar.names = subset(colnames(sample.chains),grepl("GenVar",colnames(sample.chains)))
ResidVar.names = subset(colnames(sample.chains),grepl("ResidVar",colnames(sample.chains)))
Herit.names = subset(colnames(sample.chains),grepl("Herit",colnames(sample.chains)))
GenCorr.names = subset(colnames(sample.chains),grepl("GenCorr",colnames(sample.chains)))
Struct.names = subset(colnames(sample.chains),grepl("ca12",colnames(sample.chains)))

sink("CombinedMCMCSummary_GenVar.txt")
  print(summary(sample.chains[,GenVar.names]))
  print(HPDinterval(sample.chains[,GenVar.names]))
sink()

sink("CombinedMCMCSummary_ResidVar.txt")
  print(summary(sample.chains[,ResidVar.names]))
  print(HPDinterval(sample.chains[,ResidVar.names]))
sink()

sink("CombinedMCMCSummary_Herit.txt")
  print(summary(sample.chains[,Herit.names]))
  print(HPDinterval(sample.chains[,Herit.names]))
sink()

#Only use if MTM or SEM involved
sink("CombinedMCMCSummary_GenCorr.txt")
  print(summary(sample.chains[,GenCorr.names]))
  print(HPDinterval(sample.chains[,GenCorr.names]))
sink()

#Only use if SEM involved
sink("CombinedMCMCSummary_Struct.txt")
  print(summary(sample.chains[,Struct.names]))
  print(HPDinterval(sample.chains[,Struct.names]))
sink()

################################################
### Make sure an R file with script is placed in the folder with other folders of JWAS output
### Combine Marker genotype summary information across multiple analyses into a single file:
map = read.table("Map_Ordered.csv",sep=",",header=TRUE)
fold = list.dirs()
traits = array(gsub("./results_","",fold))
traits = array(gsub("_BB","",traits))

for(i in 1:dim(array(fold))){
  out = list.files(path = fold[i],pattern="marker_effects_genotypes.txt")
  if(dim(array(out))==0){
    next
  }
  if(dim(array(out))==1){
    if(traits[i]=="MTM1" | traits[i]=="MTM2"){
        temp = read.table(paste0(fold[i],"/",out),sep=",",header=TRUE)
        sub.trait = array(unique(temp[,"Trait"]))
        for(j in dim(sub.trait)){
```

```
        abbr = gsub("[^A-Z]","",sub.trait[j])
        temp1 = temp[which(temp$Trait==sub.trait[j]),]
        name.temp = colnames(temp1[,3:5])
        new.names = paste0(traits[i],"_",abbr,"_",name.temp)
        names(temp1)[names(temp1) %in% name.temp] = new.names
        map = merge(map,temp1[,2:5],by.x="markerID",by.y="Marker_ID")
        }
    }else{
        temp = read.table(paste0(fold[i],"/",out),sep=",",header=TRUE)
        name.temp = colnames(temp[,3:5])
        new.names = paste0(traits[i],"_",name.temp)
        names(temp)[names(temp) %in% name.temp] = new.names
        map = merge(map,temp[,2:5],by.x="markerID",by.y="Marker_ID")
    }
  }
  if(traits[i] == "SEM1" | traits[i] == "SEM2"){
    for(k in 1:dim(array(out))){
      type = ifelse(grepl("indirect",out[k])==TRUE,"i",ifelse(grepl("overall",out[k])==TRUE,"o",ifelse(grepl("direct",out[k])==TRUE,"d","c")))
      temp = read.table(paste0(fold[i],"/",out[k]),sep=",",header=TRUE)
      sub.trait = array(unique(temp[,"Trait"]))
      for(j in dim(sub.trait)){
        abbr = gsub("[^A-Z]","",sub.trait[j])
        temp1 = temp[which(temp$Trait==sub.trait[j]),]
        name.temp = colnames(temp1[,3:5])
        new.names = paste0(traits[i],"_",type,abbr,"_",name.temp)
        names(temp1)[names(temp1) %in% name.temp] = new.names
        map = merge(map,temp1[,2:5],by.x="markerID",by.y="Marker_ID")
      }
    }
  }
}

write.table(map,"CombinedMarkerEffectsGenotypes.txt",sep=",",row.names=FALSE,quote=FALSE)
remove(temp,i,name.temp,new.names,out) #clean up environment

################################################
### Make sure an R file with script is placed in the folder with other folders of JWAS output
### Combine GWAS window information across multiple analyses into a single file:
map = read.table("Windows.txt",header=TRUE)
map.names = colnames(map)

#Find folders & traits to parse information from:
fold = list.dirs()
traits = array(gsub("./results_","",fold))
traits = array(gsub("_BB","",traits))

for(i in 1:dim(array(fold))){
  out = list.files(path = fold[i],pattern="^GWAS")
  if(dim(array(out))==0){
    next
  }
  if(dim(array(out))==1){
    temp = read.table(paste0(fold[i],"/",out),sep=",",header=TRUE)
    name.temp = colnames(temp[,9:13])
    new.names = paste0(traits[i],"_",name.temp)
    names(temp)[names(temp) %in% name.temp] = new.names
    map = merge(map,temp[,2:13],by=map.names)
  }
  if(traits[i]=="MTM1" | traits[i]=="MTM2"){
    for(j in 1:dim(array(out))){
      sub.trait = gsub("[^A-Z]","",gsub("GWAS_MCMC_","",out[j]))
      temp = read.table(paste0(fold[i],"/",out[j]),sep=",",header=TRUE)
      name.temp = colnames(temp[,9:13])
      new.names = paste0(traits[i],"_",sub.trait,"_",name.temp)
      names(temp)[names(temp) %in% name.temp] = new.names
      map = merge(map,temp[,2:13],by=map.names)
```

```
    }
  }
  if(traits[i] == "SEM1" | traits[i] == "SEM2"){
    for(j in 1:dim(array(out))){
      type = ifelse(grepl("indirect",out[j])==TRUE,"i",ifelse(grepl("overall",out[j])==TRUE,"o","d"))
      sub.trait = gsub("[^A-Z]","",gsub("GWAS_MCMC","",out[j]))
      temp = read.table(paste0(fold[i],"/",out[j]),sep=",",header=TRUE)
      name.temp = colnames(temp[,9:13])
      new.names = paste0(traits[i],"_",type,sub.trait,"_",name.temp)
      names(temp)[names(temp) %in% name.temp] = new.names
      map = merge(map,temp[,2:13],by=map.names)
    }
  }
}
map_ordered = map[order(map$window),]
write.table(map_ordered,"CombinedGWAS.txt",sep=",",row.names=FALSE,quote=FALSE)

##################################################
### Make sure an R file with script is placed in the folder with combined JWAS output

###############################################
########## MCMC Summary/Examples #############
###############################################

#Read dat in across multiple analysis folders
samples.pi001 = read.table("./pi001/CombinedMCMCSamples.txt",sep=",",header=TRUE)
samples.pi90 = read.table("./pi90/CombinedMCMCSamples.txt",sep=",",header=TRUE)
samples.pi95 = read.table("./pi95/CombinedMCMCSamples.txt",sep=",",header=TRUE)

##################################################
##Autocorrelation
library(coda)
options(scipen = 999)
traits = array(c("AFC","BC","BS1","BS2","iBWT","iDENS","iIMF","iYG","OS","YG","MTM1","MTM2","SEM1","SEM2"))
sample.chains = as.mcmc(samples.pi90[,2:dim(samples.pi90)[2]])

sink("Autocorrelation_pi90.txt")
for(t in 1:dim(traits)){
  trait.names = array(subset(colnames(sample.chains),startsWith(colnames(sample.chains),traits[t])))
  for(i in 1:dim(array(trait.names))){
    print(trait.names[i])
    print(autocorr(sample.chains[,trait.names[i]],lags = c(0,1,5,10,50,100)))
  }
}
sink()

##################################################
## Figure 2:
## Heritability density plots grouped by trait across analyses and pi
par(mfrow=c(2,4),mar=c(2,3,1.5,1.5))
xrange = c(0,1)
yrange = c(0,10)

#iBWT
plot(xrange,yrange,yaxt="n",xaxt="n",xlab="",ylab="",cex.lab=1,type="n")
abline(h=c(seq(0,10,by=1)),v=c(seq(0,1,by=0.05)),col="lightgray",lwd=1.5,lty="dashed")
lines(density(samples.pi001[,"iBWT001_Herit"]),lty="solid",col="black",lwd=1.5)
lines(density(samples.pi90[,"iBWT90_Herit"]),lty="dashed",col="blue",lwd=1.5)
lines(density(samples.pi95[,"iBWT95_Herit"]),lty="dotdash",col="red",lwd=1.5)

legend("topleft",legend=c(expression(paste(pi," = 0.001")),expression(paste(pi," = 0.900")),expression(paste(pi," = 0.950"))),
       col=c("black","blue","red"),lty=c(1,2,4),lwd=1.5)
axis(2,at=seq(0,10,by=1),las=1,cex.axis=0.9)
```

```
mtext("A)",side=3,line=0,adj=0,font=2,cex=0.9)

#AFC
plot(xrange,yrange,yaxt="n",xaxt="n",xlab="Heritability",ylab="Density",cex.lab=1,type="n")
abline(h=c(seq(0,10,by=1)),v=c(seq(0,1,by=0.05)),col="lightgray",lwd=1.5,lty="dashed")
lines(density(samples.pi001[,"AFC001_Herit"]),lty="solid",col="black",lwd=1.5)
lines(density(samples.pi90[,"AFC90_Herit"]),lty="dashed",col="blue",lwd=1.5)
lines(density(samples.pi95[,"AFC95_Herit"]),lty="dotdash",col="red",lwd=1.5)

legend("topleft",legend=c(expression(paste(pi," = 0.001")),expression(paste(pi," = 0.900")),expression(paste(pi," = 0.950"))),
       col=c("black","blue","red"),lty=c(1,2,4),lwd=1.5)
mtext("B)",side=3,line=0,adj=0,font=2,cex=0.9)

#iYG +YG
plot(xrange,yrange,yaxt="n",xaxt="n",xlab="Heritability",ylab="Density",cex.lab=1,type="n")
abline(h=c(seq(0,10,by=1)),v=c(seq(0,1,by=0.05)),col="lightgray",lwd=1.5,lty="dashed")
lines(density(samples.pi001[,"iYG001_Herit"]),lty="solid",col="black",lwd=1.5)
lines(density(samples.pi90[,"iYG90_Herit"]),lty="dashed",col="blue",lwd=1.5)
lines(density(samples.pi95[,"iYG95_Herit"]),lty="dotdash",col="red",lwd=1.5)
lines(density(samples.pi001[,"YG001_Herit"]),lty="solid",col="green",lwd=1.5)
lines(density(samples.pi90[,"YG90_Herit"]),lty="dashed",col="purple",lwd=1.5)
lines(density(samples.pi95[,"YG95_Herit"]),lty="dotdash",col="black",lwd=1.5)

legend("topleft",legend=c(expression(paste("Rep., ",pi," = 0.001")),
                          expression(paste("Rep., ",pi," = 0.900")),
                          expression(paste("Rep., ",pi," = 0.950")),
                          expression(paste("UBT, ",pi," = 0.001")),
                          expression(paste("UBT, ",pi," = 0.900")),
                          expression(paste("UBT, ",pi," = 0.950"))),
       col=c("black","blue","red","green","purple"),lty=c(1,2,4),lwd=1.5)
mtext("C)",side=3,line=0,adj=0,font=2,cex=0.9)

#iDENS
plot(xrange,yrange,yaxt="n",xaxt="n",xlab="Heritability",ylab="Density",cex.lab=1,type="n")
abline(h=c(seq(0,10,by=1)),v=c(seq(0,1,by=0.05)),col="lightgray",lwd=1.5,lty="dashed")
lines(density(samples.pi001[,"iDENS001_Herit"]),lty="solid",col="black",lwd=1.5)
lines(density(samples.pi90[,"iDENS90_Herit"]),lty="dashed",col="blue",lwd=1.5)
lines(density(samples.pi95[,"iDENS95_Herit"]),lty="dotdash",col="red",lwd=1.5)

legend("topleft",legend=c(expression(paste(pi," = 0.001")),expression(paste(pi," = 0.900")),expression(paste(pi," = 0.950"))),
       col=c("black","blue","red"),lty=c(1,2,4),lwd=1.5)
mtext("D)",side=3,line=0,adj=0,font=2,cex=0.9)

#iIMF
plot(xrange,yrange,yaxt="n",xaxt="n",xlab="Heritability",ylab="Density",cex.lab=1,type="n")
abline(h=c(seq(0,10,by=1)),v=c(seq(0,1,by=0.05)),col="lightgray",lwd=1.5,lty="dashed")
lines(density(samples.pi001[,"iIMF001_Herit"]),lty="solid",col="black",lwd=1.5)
lines(density(samples.pi90[,"iIMF90_Herit"]),lty="dashed",col="blue",lwd=1.5)
lines(density(samples.pi95[,"iIMF95_Herit"]),lty="dotdash",col="red",lwd=1.5)

legend("topleft",legend=c(expression(paste(pi," = 0.001")),expression(paste(pi," = 0.900")),expression(paste(pi," = 0.950"))),
       col=c("black","blue","red"),lty=c(1,2,4),lwd=1.5)
axis(2,at=seq(0,10,by=1),las=1,cex.axis=0.9)
axis(1,at=seq(0,1,by=0.1))
mtext("E)",side=3,line=0,adj=0,font=2,cex=0.9)

#BS
plot(xrange,yrange,yaxt="n",xaxt="n",xlab="Heritability",ylab="Density",cex.lab=1,type="n")
abline(h=c(seq(0,10,by=1)),v=c(seq(0,1,by=0.05)),col="lightgray",lwd=1.5,lty="dashed")
lines(density(samples.pi001[,"BS1001_Herit"]),lty="solid",col="black",lwd=1.5)
lines(density(samples.pi90[,"BS190_Herit"]),lty="dashed",col="blue",lwd=1.5)
```

```r
lines(density(samples.pi95[,"BS195_Herit"]),lty="dotdash",col="red",lwd=1.5)
lines(density(samples.pi001[,"BS2001_Herit"]),lty="solid",col="green",lwd=1.5)
lines(density(samples.pi90[,"BS290_Herit"]),lty="dashed",col="purple",lwd=1.5)
lines(density(samples.pi95[,"BS295_Herit"]),lty="dotdash",col="black",lwd=1.5)
lines(density(samples.pi90[,"MTM190_BS_Herit"]),lty="solid",col="blue",lwd=1.5)
lines(density(samples.pi90[,"MTM290_BS_Herit"]),lty="dashed",col="red",lwd=1.5)
lines(density(samples.pi90[,"SEM190_BS_Herit"]),lty="dotdash",col="green",lwd=1.5)
lines(density(samples.pi90[,"SEM290_BS_Herit"]),lty="solid",col="purple",lwd=1.5)


legend("topleft",legend=c(expression(paste("Model 1 Uni., ",pi," = 0.001")),
                          expression(paste("Model 1 Uni., ",pi," = 0.900")),
                          expression(paste("Model 1 Uni., ",pi," = 0.950")),
                          expression(paste("Model 2 Uni., ",pi," = 0.001")),
                          expression(paste("Model 2 Uni., ",pi," = 0.900")),
                          expression(paste("Model 2 Uni., ",pi," = 0.950")),
                          expression(paste("Model 1 MTM, ",pi," = 0.900")),
                          expression(paste("Model 2 MTM, ",pi," = 0.900")),
                          expression(paste("Model 1 SEM, ",pi," = 0.900")),
                          expression(paste("Model 2 SEM, ",pi," = 0.900"))),
       col=c("black","blue","red","green","purple"),lty=c(1,2,4),lwd=1.5)
axis(1,at=seq(0,1,by=0.1))
mtext("F)",side=3,line=0,adj=0,font=2,cex=0.9)

#BC
plot(xrange,yrange,yaxt="n",xaxt="n",xlab="Heritability",ylab="Density",cex.lab=1,type="n")
abline(h=c(seq(0,10,by=1)),v=c(seq(0,1,by=0.05)),col="lightgray",lwd=1.5,lty="dashed")
lines(density(samples.pi001[,"BC001_Herit"]),lty="solid",col="black",lwd=1.5)
lines(density(samples.pi90[,"BC90_Herit"]),lty="dashed",col="blue",lwd=1.5)
lines(density(samples.pi95[,"BC95_Herit"]),lty="dotdash",col="red",lwd=1.5)
lines(density(samples.pi90[,"MTM190_BC_Herit"]),lty="solid",col="green",lwd=1.5)
lines(density(samples.pi90[,"SEM190_BC_Herit"]),lty="dashed",col="purple",lwd=1.5)

legend("topleft",legend=c(expression(paste("Model 1 Uni., ",pi," = 0.001")),
                          expression(paste("Model 1 Uni., ",pi," = 0.900")),
                          expression(paste("Model 1 Uni., ",pi," = 0.950")),
                          expression(paste("Model 1 MTM, ",pi," = 0.900")),
                          expression(paste("Model 1 SEM, ",pi," = 0.900"))),
       col=c("black","blue","red","green","purple"),lty=c(1,2,4),lwd=1.5)
axis(1,at=seq(0,1,by=0.1))
mtext("G)",side=3,line=0,adj=0,font=2,cex=0.9)

#OS
plot(xrange,yrange,yaxt="n",xaxt="n",xlab="Heritability",ylab="Density",cex.lab=1,type="n")
abline(h=c(seq(0,10,by=1)),v=c(seq(0,1,by=0.05)),col="lightgray",lwd=1.5,lty="dashed")
lines(density(samples.pi001[,"OS001_Herit"]),lty="solid",col="black",lwd=1.5)
lines(density(samples.pi90[,"OS90_Herit"]),lty="dashed",col="blue",lwd=1.5)
lines(density(samples.pi95[,"OS95_Herit"]),lty="dotdash",col="red",lwd=1.5)
lines(density(samples.pi90[,"MTM290_OS_Herit"]),lty="solid",col="green",lwd=1.5)
lines(density(samples.pi90[,"SEM290_OS_Herit"]),lty="dashed",col="purple",lwd=1.5)

legend("topleft",legend=c(expression(paste("Model 2 Uni., ",pi," = 0.001")),
                          expression(paste("Model 2 Uni., ",pi," = 0.900")),
                          expression(paste("Model 2 Uni., ",pi," = 0.950")),
                          expression(paste("Model 2 MTM, ",pi," = 0.900")),
                          expression(paste("Model 2 SEM, ",pi," = 0.900"))),
       col=c("black","blue","red","green","purple"),lty=c(1,2,4),lwd=1.5)
axis(1,at=seq(0,1,by=0.1))
mtext("H)",side=3,line=0,adj=0,font=2,cex=0.9)

##############################################
## Figure 3:
## Genetic Correlations & Causal Structure
par(mfrow=c(1,2),mar=c(2,3,1.5,1.5))
xrange = c(-1,1)
yrange = c(0,4)
```

```
#Model 1
plot(xrange,yrange,yaxt="n",xaxt="n",xlab="",ylab="",cex.lab=1,type="n")
abline(h=c(seq(0,4,by=1)),v=c(seq(-1,1,by=0.05)),col="lightgray",lwd=1.5,lty="dashed")
lines(density(samples.pi90[,"MTM190_GenCorr"]),lty="solid",col="black",lwd=1.5)
lines(density(samples.pi90[,"SEM190_GenCorr"]),lty="dashed",col="blue",lwd=1.5)
lines(density(samples.pi90[,"SEM190_ca12"]),lty="dotdash",col="red",lwd=1.5)
legend("topleft",legend=c("MTM GC","SEM GC","SEM CS"),
       col=c("black","blue","red"),lty=c(1,2,4),lwd=1.5)
axis(2,at=seq(0,4,by=1),las=1,cex.axis=0.8)
axis(1,at=seq(-1,1,by=0.1),cex.axis=0.8)
mtext("A)",side=3,line=0,adj=0,font=2,cex=0.9)

plot(xrange,yrange,yaxt="n",xaxt="n",xlab="",ylab="",cex.lab=1,type="n")
abline(h=c(seq(0,4,by=1)),v=c(seq(-1,1,by=0.05)),col="lightgray",lwd=1.5,lty="dashed")
lines(density(samples.pi90[,"MTM290_GenCorr"]),lty="solid",col="black",lwd=1.5)
lines(density(samples.pi90[,"SEM290_GenCorr"]),lty="dashed",col="blue",lwd=1.5)
lines(density(samples.pi90[,"SEM290_ca12"]),lty="dotdash",col="red",lwd=1.5)
legend("topleft",legend=c("MTM GC","SEM GC","SEM CS"),
       col=c("black","blue","red"),lty=c(1,2,4),lwd=1.5)
axis(2,at=seq(0,4,by=1),las=1,cex.axis=0.8)
axis(1,at=seq(-1,1,by=0.1),cex.axis=0.8)
mtext("B)",side=3,line=0,adj=0,font=2,cex=0.9)

##############################################
##########  GWAS Summary/Plots   #############
##############################################

#Read dat in across multiple analysis folders
gwas.pi001 = read.table("./NEW GWAS RUNS 260422 pi001/CombinedGWAS.txt",sep=",",header=TRUE)
gwas.pi90 = read.table("./NEW GWAS RUNS 260520 pi90/CombinedGWAS.txt",sep=",",header=TRUE)
gwas.pi95 = read.table("./NEW GWAS RUNS 260520 pi95/CombinedGWAS.txt",sep=",",header=TRUE)

all.col = array(c(colnames(gwas.pi001),colnames(gwas.pi90),colnames(gwas.pi95)))
WPPA.names = subset(all.col,grepl("WPPA",all.col))

nchr=29 ##29 chromosomes
nwin=dim(gwas.pi001)[1] #all have the same number of windows

Windows=matrix(0,nchr,2,dimnames=list(1:nchr,c("Min","Max")))
for(i in 1:nchr){
  Chr<-subset(gwas.pi001,chr==i)
  Windows[i,'Min']<-min(Chr[,'window'])
  Windows[i,'Max']<-max(Chr[,'window'])
}

RefC<-matrix(0,nchr,1)
for(i in 1:nchr){
  RefC[i,1]<-round(((Windows[i,'Max']-Windows[i,'Min'])/2)+Windows[i,'Min'])
} #Center points to label with

#Consistent ranges across analyses
xrange<-range(Windows)
yrange<-c(0,1)

#################################################
## Figure 4:
## Body Size - Uni + MTM
par(mfrow=c(3,1),mar=c(3,4.5,2,1))

#Model 1 - BS, different pi
plot(xrange,yrange,yaxt="n",xaxt="n",xlab="",ylab=expression(PPA[italic(w)]),cex.lab=1.5,type="n")
points(gwas.pi001[,'window'],gwas.pi001[,'BS1001_WPPA'],pch=8,cex=1.25,col="black")
points(gwas.pi90[,'window'],gwas.pi90[,'BS190_WPPA'],pch=16,cex=1.25,col="blue")
points(gwas.pi95[,'window'],gwas.pi95[,'BS195_WPPA'],pch=6,cex=1.25,col="red")
axis(2,las=1)
```

```
axis(1,at=c(Windows[,1],2501),labels=c("","","","","","","","","","","","","","","","","","","","","","","","","","","","","",""),tck=0.03)
axis(1,at=c(Windows[,1],2501),labels=c("","","","","","","","","","","","","","","","","","","","","","","","","","","","","",""),tck=-0.03)
axis(1,at=RefC[,1],labels=c(1,2,3,4,5,6,7,8,9,10,11,12,13,14,15,16,17,18,19,20,21,22,23,24,25,26,27,28,29),tck=0,gap.axis=0.25)
abline(h=0.8,lty=2,col="grey30")
legend("topright", legend=c(expression(paste(pi," = 0.001")),expression(paste(pi," = 0.900")),expression(paste(pi," = 0.950"))),
       col=c("black","blue","red"),pch=c(8,16,6))
mtext("A)",side=3,line=0.5,adj=0,font=2)

#Model 2 - BS, different pi
plot(xrange,yrange,yaxt="n",xaxt="n",xlab="",ylab=expression(PPA[italic(w)]),cex.lab=1.5,type="n")
points(gwas.pi001[,'window'],gwas.pi001[,'BS2001_WPPA'],pch=8,cex=1.25,col="black")
points(gwas.pi90[,'window'],gwas.pi90[,'BS290_WPPA'],pch=16,cex=1.25,col="blue")
points(gwas.pi95[,'window'],gwas.pi95[,'BS295_WPPA'],pch=6,cex=1.25,col="red")
axis(2,las=1)
axis(1,at=c(Windows[,1],2501),labels=c("","","","","","","","","","","","","","","","","","","","","","","","","","","","","",""),tck=0.03)
axis(1,at=c(Windows[,1],2501),labels=c("","","","","","","","","","","","","","","","","","","","","","","","","","","","","",""),tck=-0.03)
axis(1,at=RefC[,1],labels=c(1,2,3,4,5,6,7,8,9,10,11,12,13,14,15,16,17,18,19,20,21,22,23,24,25,26,27,28,29),tck=0,gap.axis=0.25)
abline(h=0.8,lty=2,col="grey30")
legend("topright", legend=c(expression(paste(pi," = 0.001")),expression(paste(pi," = 0.900")),expression(paste(pi," = 0.950"))),
       col=c("black","blue","red"),pch=c(8,16,6))
mtext("B)",side=3,line=0.5,adj=0,font=2)

#Model 1/2 - MTM
plot(xrange,yrange,yaxt="n",xaxt="n",xlab="",ylab=expression(PPA[italic(w)]),cex.lab=1.5,type="n")
points(gwas.pi90[,'window'],gwas.pi90[,'MTM190_BS_WPPA'],pch=8,cex=1.25,col="black")
points(gwas.pi90[,'window'],gwas.pi90[,'MTM290_BS_WPPA'],pch=16,cex=1.25,col="blue")
axis(2,las=1)
axis(1,at=c(Windows[,1],2501),labels=c("","","","","","","","","","","","","","","","","","","","","","","","","","","","","",""),tck=0.03)
axis(1,at=c(Windows[,1],2501),labels=c("","","","","","","","","","","","","","","","","","","","","","","","","","","","","",""),tck=-0.03)
axis(1,at=RefC[,1],labels=c(1,2,3,4,5,6,7,8,9,10,11,12,13,14,15,16,17,18,19,20,21,22,23,24,25,26,27,28,29),tck=0,gap.axis=0.25)
abline(h=0.8,lty=2,col="grey30")
legend("topright", legend=c(expression(paste("Model 1, ",pi," = 0.900")),expression(paste("Model 2, ",pi," = 0.900"))),
       col=c("black","blue","red"),pch=c(8,16,6))
mtext("C)",side=3,line=0.5,adj=0,font=2)
mtext("Chromosome",side=1,line=2)

################################################
## Figure 5:
## Body Comp/OS - Uni + MTM
par(mfrow=c(3,1),mar=c(3,4.5,2,1))

#Model 1 - BC, different pi
plot(xrange,yrange,yaxt="n",xaxt="n",xlab="",ylab=expression(PPA[italic(w)]),cex.lab=1.5,type="n")
points(gwas.pi001[,'window'],gwas.pi001[,'BC001_WPPA'],pch=8,cex=1.25,col="black")
points(gwas.pi90[,'window'],gwas.pi90[,'BC90_WPPA'],pch=16,cex=1.25,col="blue")
points(gwas.pi95[,'window'],gwas.pi95[,'BC95_WPPA'],pch=6,cex=1.25,col="red")
axis(2,las=1)
axis(1,at=c(Windows[,1],2501),labels=c("","","","","","","","","","","","","","","","","","","","","","","","","","","","","",""),tck=0.03)
axis(1,at=c(Windows[,1],2501),labels=c("","","","","","","","","","","","","","","","","","","","","","","","","","","","","",""),tck=-0.03)
axis(1,at=RefC[,1],labels=c(1,2,3,4,5,6,7,8,9,10,11,12,13,14,15,16,17,18,19,20,21,22,23,24,25,26,27,28,29),tck=0,gap.axis=0.25)
abline(h=0.8,lty=2,col="grey30")
```

```
legend("topright", legend=c(expression(paste(pi," = 0.001")),expression(paste(pi," = 0.900")),expression(paste(pi," = 0.950"))),
       col=c("black","blue","red"),pch=c(8,16,6))
mtext("A)",side=3,line=0.5,adj=0,font=2)

#Model 2 - OS, different pi
plot(xrange,yrange,yaxt="n",xaxt="n",xlab="",ylab=expression(PPA[italic(w)]),cex.lab=1.5,type="n")
points(gwas.pi001[,'window'],gwas.pi001[,'OS001_WPPA'],pch=8,cex=1.25,col="black")
points(gwas.pi90[,'window'],gwas.pi90[,'OS90_WPPA'],pch=16,cex=1.25,col="blue")
points(gwas.pi95[,'window'],gwas.pi95[,'OS95_WPPA'],pch=6,cex=1.25,col="red")
axis(2,las=1)
axis(1,at=c(Windows[,1],2501),labels=c("","","","","","","","","","","","","","","","","","","","","","","","","","","","","",""),tck=0.03)
axis(1,at=c(Windows[,1],2501),labels=c("","","","","","","","","","","","","","","","","","","","","","","","","","","","","",""),tck=-0.03)
axis(1,at=RefC[,1],labels=c(1,2,3,4,5,6,7,8,9,10,11,12,13,14,15,16,17,18,19,20,21,22,23,24,25,26,27,28,29),tck=0,gap.axis=0.25)
abline(h=0.8,lty=2,col="grey30")
legend("topright", legend=c(expression(paste(pi," = 0.001")),expression(paste(pi," = 0.900")),expression(paste(pi," = 0.950"))),
       col=c("black","blue","red"),pch=c(8,16,6))
mtext("B)",side=3,line=0.5,adj=0,font=2)

#Model 1/2 - MTM
plot(xrange,yrange,yaxt="n",xaxt="n",xlab="",ylab=expression(PPA[italic(w)]),cex.lab=1.5,type="n")
points(gwas.pi90[,'window'],gwas.pi90[,'MTM190_BC_WPPA'],pch=8,cex=1.25,col="black")
points(gwas.pi90[,'window'],gwas.pi90[,'MTM290_OS_WPPA'],pch=16,cex=1.25,col="blue")
axis(2,las=1)
axis(1,at=c(Windows[,1],2501),labels=c("","","","","","","","","","","","","","","","","","","","","","","","","","","","","",""),tck=0.03)
axis(1,at=c(Windows[,1],2501),labels=c("","","","","","","","","","","","","","","","","","","","","","","","","","","","","",""),tck=-0.03)
axis(1,at=RefC[,1],labels=c(1,2,3,4,5,6,7,8,9,10,11,12,13,14,15,16,17,18,19,20,21,22,23,24,25,26,27,28,29),tck=0,gap.axis=0.25)
abline(h=0.8,lty=2,col="grey30")
legend("topright", legend=c(expression(paste("Model 1, BC, ",pi," = 0.900")),expression(paste("Model 2, OS, ",pi," = 0.900"))),
       col=c("black","blue","red"),pch=c(8,16,6))
mtext("C)",side=3,line=0.5,adj=0,font=2)
mtext("Chromosome",side=1,line=2)

#################################################
## Figure 6:
## SEM of Model 1 and 2
par(mfrow=c(2,1),mar=c(3,4.5,2,1))

#SEM1
plot(xrange,yrange,yaxt="n",xaxt="n",xlab="",ylab=expression(PPA[italic(w)]),cex.lab=1.5,type="n")
points(gwas.pi90[,'window'],gwas.pi90[,'SEM190_oBS_WPPA'],pch=8,cex=1.25,col="black")
points(gwas.pi90[,'window'],gwas.pi90[,'SEM190_dBC_WPPA'],pch=16,cex=1.25,col="blue")
points(gwas.pi90[,'window'],gwas.pi90[,'SEM190_iBC_WPPA'],pch=6,cex=1.25,col="darkgreen")
points(gwas.pi90[,'window'],gwas.pi90[,'SEM190_oBC_WPPA'],pch=0,cex=1.25,col="red")
axis(2,las=1)
axis(1,at=c(Windows[,1],2501),labels=c("","","","","","","","","","","","","","","","","","","","","","","","","","","","","",""),tck=0.03)
axis(1,at=c(Windows[,1],2501),labels=c("","","","","","","","","","","","","","","","","","","","","","","","","","","","","",""),tck=-0.03)
axis(1,at=RefC[,1],labels=c(1,2,3,4,5,6,7,8,9,10,11,12,13,14,15,16,17,18,19,20,21,22,23,24,25,26,27,28,29),tck=0,gap.axis=0.25)
abline(h=0.8,lty=2,col="grey30")
legend("topright", legend=c("overall BS","direct BC","indirect BC","overall BC"),
       col=c("black","blue","darkgreen","red"),pch=c(8,16,6,0))
mtext("A)",side=3,line=0.5,adj=0,font=2)

#SEM2
plot(xrange,yrange,yaxt="n",xaxt="n",xlab="",ylab=expression(PPA[italic(w)]),cex.lab=1.5,
```

```
type="n")
points(gwas.pi90[,'window'],gwas.pi90[,'SEM290_oBS_WPPA'],pch=8,cex=1.25,col="black")
points(gwas.pi90[,'window'],gwas.pi90[,'SEM290_dOS_WPPA'],pch=16,cex=1.25,col="blue")
points(gwas.pi90[,'window'],gwas.pi90[,'SEM290_iOS_WPPA'],pch=6,cex=1.25,col="darkgreen")
points(gwas.pi90[,'window'],gwas.pi90[,'SEM290_oOS_WPPA'],pch=0,cex=1.25,col="red")
axis(2,las=1)
axis(1,at=c(Windows[,1],2501),labels=c("","","","","","","","","","","","","","","","","","","","","","","","","","","","","",""),tck=0.03)
axis(1,at=c(Windows[,1],2501),labels=c("","","","","","","","","","","","","","","","","","","","","","","","","","","","","",""),tck=-0.03)
axis(1,at=RefC[,1],labels=c(1,2,3,4,5,6,7,8,9,10,11,12,13,14,15,16,17,18,19,20,21,22,23,24,25,26,27,28,29),tck=0,gap.axis=0.25)
abline(h=0.8,lty=2,col="grey30")
legend("topright", legend=c("overall BS","direct OS","indirect OS","overall OS"),
       col=c("black","blue","darkgreen","red"),pch=c(8,16,6,0))
mtext("B)",side=3,line=0.5,adj=0,font=2)
mtext("Chromosome",side=1,line=2)

##################################################
## Figure 7:
## Rep Traits - iBWT, AFC, iYG
par(mfrow=c(3,1),mar=c(3,4.5,2,1))

#iBWT
plot(xrange,yrange,yaxt="n",xaxt="n",xlab="",ylab=expression(PPA[italic(w)]),cex.lab=1.5,type="n")
points(gwas.pi001[,'window'],gwas.pi001[,'iBWT001_WPPA'],pch=8,cex=1.25,col="black")
points(gwas.pi90[,'window'],gwas.pi90[,'iBWT90_WPPA'],pch=16,cex=1.25,col="blue")
points(gwas.pi95[,'window'],gwas.pi95[,'iBWT95_WPPA'],pch=6,cex=1.25,col="red")
axis(2,las=1)
axis(1,at=c(Windows[,1],2501),labels=c("","","","","","","","","","","","","","","","","","","","","","","","","","","","","",""),tck=0.03)
axis(1,at=c(Windows[,1],2501),labels=c("","","","","","","","","","","","","","","","","","","","","","","","","","","","","",""),tck=-0.03)
axis(1,at=RefC[,1],labels=c(1,2,3,4,5,6,7,8,9,10,11,12,13,14,15,16,17,18,19,20,21,22,23,24,25,26,27,28,29),tck=0,gap.axis=0.25)
abline(h=0.8,lty=2,col="grey30")
legend("topright", legend=c(expression(paste(pi," = 0.001")),expression(paste(pi," = 0.900")),expression(paste(pi," = 0.950"))),
       col=c("black","blue","red"),pch=c(8,16,6))
mtext("A)",side=3,line=0.5,adj=0,font=2)

#AFC
plot(xrange,yrange,yaxt="n",xaxt="n",xlab="",ylab=expression(PPA[italic(w)]),cex.lab=1.5,type="n")
points(gwas.pi001[,'window'],gwas.pi001[,'AFC001_WPPA'],pch=8,cex=1.25,col="black")
points(gwas.pi90[,'window'],gwas.pi90[,'AFC90_WPPA'],pch=16,cex=1.25,col="blue")
points(gwas.pi95[,'window'],gwas.pi95[,'AFC95_WPPA'],pch=6,cex=1.25,col="red")
axis(2,las=1)
axis(1,at=c(Windows[,1],2501),labels=c("","","","","","","","","","","","","","","","","","","","","","","","","","","","","",""),tck=0.03)
axis(1,at=c(Windows[,1],2501),labels=c("","","","","","","","","","","","","","","","","","","","","","","","","","","","","",""),tck=-0.03)
axis(1,at=RefC[,1],labels=c(1,2,3,4,5,6,7,8,9,10,11,12,13,14,15,16,17,18,19,20,21,22,23,24,25,26,27,28,29),tck=0,gap.axis=0.25)
abline(h=0.8,lty=2,col="grey30")
legend("topright", legend=c(expression(paste(pi," = 0.001")),expression(paste(pi," = 0.900")),expression(paste(pi," = 0.950"))),
       col=c("black","blue","red"),pch=c(8,16,6))
mtext("B)",side=3,line=0.5,adj=0,font=2)

#iYG
plot(xrange,yrange,yaxt="n",xaxt="n",xlab="",ylab=expression(PPA[italic(w)]),cex.lab=1.5,type="n")
points(gwas.pi001[,'window'],gwas.pi001[,'iYG001_WPPA'],pch=8,cex=1.25,col="black")
points(gwas.pi90[,'window'],gwas.pi90[,'iYG90_WPPA'],pch=16,cex=1.25,col="blue")
points(gwas.pi95[,'window'],gwas.pi95[,'iYG95_WPPA'],pch=6,cex=1.25,col="red")
axis(2,las=1)
axis(1,at=c(Windows[,1],2501),labels=c("","","","","","","","","","","","","","","","",""
```

```
,"","","","","","","","","","","","",""),tck=0.03)
axis(1,at=c(Windows[,1],2501),labels=c("","","","","","","","","","","","","","","","","","","","","","","","","","","","",""),tck=-0.03)
axis(1,at=RefC[,1],labels=c(1,2,3,4,5,6,7,8,9,10,11,12,13,14,15,16,17,18,19,20,21,22,23,24,25,26,27,28,29),tck=0,gap.axis=0.25)
abline(h=0.8,lty=2,col="grey30")
legend("topright", legend=c(expression(paste(pi," = 0.001")),expression(paste(pi," = 0.900")),expression(paste(pi," = 0.950"))),
       col=c("black","blue","red"),pch=c(8,16,6))
mtext("C)",side=3,line=0.5,adj=0,font=2)
mtext("Chromosome",side=1,line=2)

##################################################
## Figure 8:
## NCERT +UBT - iDENS, iIMF, YG
par(mfrow=c(3,1),mar=c(3,4.5,2,1))

#DENS
plot(xrange,yrange,yaxt="n",xaxt="n",xlab="",ylab=expression(PPA[italic(w)]),cex.lab=1.5,type="n")
points(gwas.pi001[,'window'],gwas.pi001[,'iDENS001_WPPA'],pch=8,cex=1.25,col="black")
points(gwas.pi90[,'window'],gwas.pi90[,'iDENS90_WPPA'],pch=16,cex=1.25,col="blue")
points(gwas.pi95[,'window'],gwas.pi95[,'iDENS95_WPPA'],pch=6,cex=1.25,col="red")
axis(2,las=1)
axis(1,at=c(Windows[,1],2501),labels=c("","","","","","","","","","","","","","","","","","","","","","","","","","","","",""),tck=0.03)
axis(1,at=c(Windows[,1],2501),labels=c("","","","","","","","","","","","","","","","","","","","","","","","","","","","",""),tck=-0.03)
axis(1,at=RefC[,1],labels=c(1,2,3,4,5,6,7,8,9,10,11,12,13,14,15,16,17,18,19,20,21,22,23,24,25,26,27,28,29),tck=0,gap.axis=0.25)
abline(h=0.8,lty=2,col="grey30")
legend("topright", legend=c(expression(paste(pi," = 0.001")),expression(paste(pi," = 0.900")),expression(paste(pi," = 0.950"))),
       col=c("black","blue","red"),pch=c(8,16,6))
mtext("A)",side=3,line=0.5,adj=0,font=2)

#IMF
plot(xrange,yrange,yaxt="n",xaxt="n",xlab="",ylab=expression(PPA[italic(w)]),cex.lab=1.5,type="n")
points(gwas.pi001[,'window'],gwas.pi001[,'iIMF001_WPPA'],pch=8,cex=1.25,col="black")
points(gwas.pi90[,'window'],gwas.pi90[,'iIMF90_WPPA'],pch=16,cex=1.25,col="blue")
points(gwas.pi95[,'window'],gwas.pi95[,'iIMF95_WPPA'],pch=6,cex=1.25,col="red")
axis(2,las=1)
axis(1,at=c(Windows[,1],2501),labels=c("","","","","","","","","","","","","","","","","","","","","","","","","","","","",""),tck=0.03)
axis(1,at=c(Windows[,1],2501),labels=c("","","","","","","","","","","","","","","","","","","","","","","","","","","","",""),tck=-0.03)
axis(1,at=RefC[,1],labels=c(1,2,3,4,5,6,7,8,9,10,11,12,13,14,15,16,17,18,19,20,21,22,23,24,25,26,27,28,29),tck=0,gap.axis=0.25)
abline(h=0.8,lty=2,col="grey30")
legend("topright", legend=c(expression(paste(pi," = 0.001")),expression(paste(pi," = 0.900")),expression(paste(pi," = 0.950"))),
       col=c("black","blue","red"),pch=c(8,16,6))
mtext("B)",side=3,line=0.5,adj=0,font=2)

#YG
plot(xrange,yrange,yaxt="n",xaxt="n",xlab="",ylab=expression(PPA[italic(w)]),cex.lab=1.5,type="n")
points(gwas.pi001[,'window'],gwas.pi001[,'YG001_WPPA'],pch=8,cex=1.25,col="black")
points(gwas.pi90[,'window'],gwas.pi90[,'YG90_WPPA'],pch=16,cex=1.25,col="blue")
points(gwas.pi95[,'window'],gwas.pi95[,'YG95_WPPA'],pch=6,cex=1.25,col="red")
axis(2,las=1)
axis(1,at=c(Windows[,1],2501),labels=c("","","","","","","","","","","","","","","","","","","","","","","","","","","","",""),tck=0.03)
axis(1,at=c(Windows[,1],2501),labels=c("","","","","","","","","","","","","","","","","","","","","","","","","","","","",""),tck=-0.03)
axis(1,at=RefC[,1],labels=c(1,2,3,4,5,6,7,8,9,10,11,12,13,14,15,16,17,18,19,20,21,22,23,24,25,26,27,28,29),tck=0,gap.axis=0.25)
abline(h=0.8,lty=2,col="grey30")
```

```r
legend("topright", legend=c(expression(paste(pi," = 0.001")),expression(paste(pi," = 0.900")),expression(paste(pi," = 0.950"))),
       col=c("black","blue","red"),pch=c(8,16,6))
mtext("C)",side=3,line=0.5,adj=0,font=2)
mtext("Chromosome",side=1,line=2)


##################################################
## Find regions of interest across analyses. Given sample size, setting threshold WPPA >=0.5 to see combined counts
WPPAComb = merge(merge(gwas.pi001,gwas.pi90,by=colnames(gwas.pi001[,1:7])),gwas.pi95,by=colnames(gwas.pi001[,1:7]))
WPPAComb = WPPAComb[,c(colnames(WPPAComb[,1:7]),WPPA.names)]
nanalyses = (dim(WPPAComb)[2]-7)

WPPAComb$AllCount50 = rowSums(WPPAComb[,c(8:nanalyses)]>=0.5)
WPPAComb$AllP50 = WPPAComb$AllCount50/nanalyses
WPPAComb$AllCount70 = rowSums(WPPAComb[,c(8:nanalyses)]>=0.7)
WPPAComb$AllP70 = WPPAComb$AllCount70/nanalyses
WPPAComb$AllCount80 = rowSums(WPPAComb[,c(8:nanalyses)]>=0.8)
WPPAComb$AllP80 = WPPAComb$AllCount80/nanalyses

##################################################
## Figure 9: Proportion of models WPPA > = 0.7 or 0.8
par(mfrow=c(1,1))
plot(xrange,yrange,yaxt="n",xaxt="n",xlab="Chromosome",ylab="Proportion",cex.lab=1.1,type="n")
points(WPPAComb[,'window'],WPPAComb[,'AllP50'],pch=8,cex=1.25,col="black")
points(WPPAComb[,'window'],WPPAComb[,'AllP70'],pch=16,cex=1.25,col="blue")
points(WPPAComb[,'window'],WPPAComb[,'AllP80'],pch=6,cex=1.25,col="red")
axis(2,las=1)
axis(1,at=c(Windows[,1],2501),labels=c("","","","","","","","","","","","","","","","","","","","","","","","","","","","","",""),tck=0.03)
axis(1,at=c(Windows[,1],2501),labels=c("","","","","","","","","","","","","","","","","","","","","","","","","","","","","",""),tck=-0.03)
axis(1,at=RefC[,1],labels=c(1,2,3,4,5,6,7,8,9,10,11,12,13,14,15,16,17,18,19,20,21,22,23,24,25,26,27,28,29),tck=0,gap.axis=0.25)
abline(h=seq(0,1,0.1),lty=2,col="grey52")
legend("topright", legend=c(expression(PPA[italic(w)] >= 0.50),expression(PPA[italic(w)] >= 0.70),expression(PPA[italic(w)] >= 0.80)),
       col=c("black","blue","red"),pch=c(8,16,6))


##################################################
## Identifying features of interest across various WPPA thresholds
interest = WPPAComb[which(WPPAComb$AllP50>0),]
write.table(interest,"WindowsOfInterest50.txt",quote=FALSE,sep=",",row.names=FALSE)

PrGenVar.names = subset(all.col,grepl("prGenVar",all.col))
PrGenVarComb = merge(merge(gwas.pi001,gwas.pi90,by=colnames(gwas.pi001[,1:7])),gwas.pi95,by=colnames(gwas.pi001[,1:7]))
PrGenVarComb = PrGenVarComb[,c(colnames(PrGenVarComb[,1:7]),PrGenVar.names)]
interest2 = PrGenVarComb[which(PrGenVarComb$window %in% interest$window),,drop=FALSE]
interest2$avgPrGenVar = rowSums(interest2[,c(8:nanalyses)])/nanalyses
write.table(interest2,"WindowsOfInterest_prgenvar.txt",quote=FALSE,sep=",",row.names=FALSE)

features = read.delim("full_feature_table.txt", sep="\t",header=TRUE) #feature table downloaded from NCBI database
uni.feat = unique(features[,c("symbol","GeneID")])
write.table(uni.feat,"UniqueFeatures.txt",sep=",",row.names=FALSE,quote=FALSE)

#All features when WPPA >=0.5
ichr = array(unique(interest$chr))
for(i in ichr){
  temp = features[which(features$chromosome==i),]
  temp2 = interest[which(interest$chr ==i),]
```

```
  if(dim(temp2)[1]>1){
    iFeatures = temp[which(temp$start >= min(temp2$wStart) & temp$end < max(temp2$wEnd)),]
  }else{
    iFeatures = temp[which(temp$start >= temp2$wStart & temp$end < temp2$wEnd),]
  }
  if(i == ichr[1]){
    write.table(iFeatures,"FeaturesOfInterest.txt",sep=",",row.names=FALSE,quote=FALSE)

  }else{write.table(iFeatures,"FeaturesOfInterest.txt",append=TRUE,sep=",",col.names=FALSE,row.names=FALSE,quote=FALSE)}
}

uni.ifeat50 = unique(iFeatures[,c("symbol","GeneID")])
write.table(uni.ifeat50,"UniqueiFeatures50.txt",sep=",",row.names=FALSE,quote=FALSE)

#All features when WPPA >=0.7
interest = WPPAComb[which(WPPAComb$AllP70>0),]
ichr = array(unique(interest$chr))
for(i in ichr){
  temp = features[which(features$chromosome==i),]
  temp2 = interest[which(interest$chr ==i),]
  if(dim(temp2)[1]>1){
    iFeatures = temp[which(temp$start >= min(temp2$wStart) & temp$end < max(temp2$wEnd)),]
  }else{
    iFeatures = temp[which(temp$start >= temp2$wStart & temp$end < temp2$wEnd),]
  }
  if(i == ichr[1]){
    write.table(iFeatures,"FeaturesOfInterest70.txt",sep=",",row.names=FALSE,quote=FALSE)

  }else{write.table(iFeatures,"FeaturesOfInterest70.txt",append=TRUE,sep=",",col.names=FALSE,row.names=FALSE,quote=FALSE)}
}
uni.ifeat70 = unique(iFeatures[,c("symbol","GeneID")])
write.table(uni.ifeat70,"UniqueiFeatures70.txt",sep=",",row.names=FALSE,quote=FALSE)

#All features when WPPA >=0.8
interest = WPPAComb[which(WPPAComb$AllP80>0),]
ichr = array(unique(interest$chr))
for(i in ichr){
  temp = features[which(features$chromosome==i),]
  temp2 = interest[which(interest$chr ==i),]
  if(dim(temp2)[1]>1){
    iFeatures = temp[which(temp$start >= min(temp2$wStart) & temp$end < max(temp2$wEnd)),]
  }else{
    iFeatures = temp[which(temp$start >= temp2$wStart & temp$end < temp2$wEnd),]
  }
}

uni.ifeat80 = unique(iFeatures[,c("symbol","GeneID")])
write.table(uni.ifeat80,"UniqueiFeatures80.txt",sep=",",row.names=FALSE,quote=FALSE)
## Caution - features names often have commas, spaces, and table is tab deliminated,
## so effort is still needed to ensure proper alignment of feature symbols to avoid errors.
```
